# Salicylic acid regulates *PIN2* auxin transporter hyperclustering and root gravitropic growth via *Remorin*‐dependent lipid nanodomain organisation in *Arabidopsis thaliana*

**DOI:** 10.1111/nph.16915

**Published:** 2020-09-30

**Authors:** Meiyu Ke, Zhiming Ma, Deyan Wang, Yanbiao Sun, Chenjin Wen, Dingquan Huang, Zichen Chen, Liang Yang, Shutang Tan, Ruixi Li, Jiří Friml, Yansong Miao, Xu Chen

**Affiliations:** ^1^ College of Life Science and Fujian Provincial Key Laboratory of Haixia Applied Plant Systems Biology Fujian Agriculture and Forestry University Fuzhou 350002 China; ^2^ Haixia Institute of Science and Technology Horticultural Plant Biology and Metabolomics Centre Fujian Agriculture and Forestry University Fuzhou 350002 China; ^3^ School of Biological Sciences Nanyang Technological University Singapore 637551 Singapore; ^4^ Singapore Centre for Environmental Life Sciences Engineering Nanyang Technological University Singapore 637551 Singapore; ^5^ Institute of Science and Technology Austria (IST Austria) Am Campus 1 Klosterneuburg 3400 Austria; ^6^ Department of Biology Southern University of Science and Technology Shenzhen 518055 China

**Keywords:** *Arabidopsis*, auxin transport, gravitropic growth, nanodomain, PIN2 hyperclustering, remorin, salicylic acid

## Abstract

- To adapt to the diverse array of biotic and abiotic cues, plants have evolved sophisticated mechanisms to sense changes in environmental conditions and modulate their growth. Growth‐promoting hormones and defence signalling fine tune plant development antagonistically. During host–pathogen interactions, this defence–growth trade‐off is mediated by the counteractive effects of the defence hormone salicylic acid (SA) and the growth hormone auxin.
- Here we revealed an underlying mechanism of SA regulating auxin signalling by constraining the plasma membrane dynamics of PIN2 auxin efflux transporter in *Arabidopsis thaliana* roots.
- The lateral diffusion of PIN2 proteins is constrained by SA signalling, during which PIN2 proteins are condensed into hyperclusters depending on REM1.2‐mediated nanodomain compartmentalisation. Furthermore, membrane nanodomain compartmentalisation by SA or Remorin (REM) assembly significantly suppressed clathrin‐mediated endocytosis. Consequently, SA‐induced heterogeneous surface condensation disrupted asymmetric auxin distribution and the resultant gravitropic response.
- Our results demonstrated a defence–growth trade‐off mechanism by which SA signalling crosstalked with auxin transport by concentrating membrane‐resident PIN2 into heterogeneous compartments.

## Introduction

Plants perceive a wide array of intracellular and extracellular chemical cues including hormones, metabolites and microbial molecules. Once they have encountered pathogenic signals, plants respond rapidly by remodelling intracellular processes and rebalancing the utilisation of limited resources between growth and defence responses, namely a growth–defence trade‐off (Huot *et al*., 2014; Guo *et al*., 2018; Lakehal *et al*., 2019; Ye *et al*., 2019). Both plant development and defence mechanisms actively involve different phytohormones that coordinate the growth–defence trade‐off during host–pathogen interactions (Huot *et al*., 2014; Lakehal *et al*., 2019; Liao *et al*., 2020). Pathogen invasion triggers the accumulation of defence hormone salicylic acid (SA) in plants that activate defence mechanisms, but this leads to the retardation of growth, including root development (Xu *et al*., 2017; Pasternak *et al*., 2019; Tan *et al*., 2019). When an exogenously applied SA reaches a concentration greater than 50 µM, it protects the plant from a broad spectrum of pathogens by activating systemic acquired resistance (Mur *et al*., 2006; Fu & Dong, 2013). Conversely, plant organ development requires the establishment of local auxin maxima, as well as a gradient distribution, such as the local auxin gradient in the root tip, in determining root architecture (Van Norman *et al*., 2013; Zhao, 2018; Lakehal *et al*., 2019). During plant immune responses, both SA and auxin signalling are regulated spatiotemporally, and are involved in orchestrating diverse defence and developmental processes. Transcriptional regulation has been shown to participate in the crosstalk between SA and auxin signalling. SA suppresses the expression of core components of auxin signalling, the *Transport Inhibitor Response 1* (*TIR1*)/*Auxin Signalling F‐box* (*AFB*) genes, which results in a stabilisation of auxin (AUX)‐inducible/indole acetic acid (IAA)‐inducible repressor proteins, and therefore attenuates auxin signalling (Wang *et al*., 2007; Pasternak *et al*., 2019). Recently, the Protein Phosphatase 2A (PP2A) complex was found to be one of the mediators involved in the phosphorylation of auxin efflux carrier PIN‐FORMED2 (PIN2) that is modulated by SA signalling (Michniewicz *et al*., 2007; Tan *et al*., 2019). However, the key molecular mechanisms underlying the crosstalk between auxin transport and SA signalling are still not fully understood.

During root development, an appropriate auxin gradient specifies the sites of organ initiation and establishes an apical–basal axis of cell polarity (Pan *et al*., 2015). The auxin gradient in a specific tissue is determined by polar auxin transport, which is strictly controlled by membrane‐resident auxin transporter PIN proteins (Adamowski & Friml, 2015). Most PINs are distributed on the plasma membrane (PM) in a polarised manner, providing a means of directing polar auxin efflux. To maintain an efficient auxin turnover, plants continuously internalise PIN from the cell surface via clathrin‐mediated endocytosis (CME). Subsequently, PIN recycles to the PM through an exocytosis system (Adamowski & Friml, 2015). PIN proteins have been previously identified to have two different populations on the PM, a less mobile form and a free diffusive pool (Kleine‐Vehn *et al*., 2011). Such heterogeneous distribution of PIN is potentially perturbed by membrane heterogeneity and interactions with distinct surface biomolecules (Kleine‐Vehn *et al*., 2011; Ott, 2017; Mamode Cassim *et al*., 2019). During pathogen–host interaction, the plant accumulates defence molecule SA that shows inhibitory effects in PIN endocytosis and influences PIN‐modulated root architecture in a concentration‐dependent manner (Du *et al*., 2013; Zhao *et al*., 2015; Pasternak *et al*., 2019), suggesting the SA‐mediated regulation of PIN turnover.

SA signalling in defence mechanisms is well known to be controlled by Nonexpressor of pathogenesis‐related genes 1 (NPR1), NPR3 and NPR4 receptors (Spoel *et al*., 2009; Fu *et al*., 2012). However, under SA treatment, *npr1* and *npr3 npr4* mutants showed comparable phenotypes compared with wild‐type in inhibiting PIN endocytosis (Du *et al*., 2013). Consistently, *npr1* was still found to respond to SA stimulation on gravitropism by showing agravitropic roots (Tan *et al*., 2019). These results implied an NPR receptor‐independent mechanism for auxin transport during SA signalling. SA was recently proven to induce lipid nanodomain compartmentalisation and increase the lipid order (Lo) phase of the PM in a remorin (REM) assembly‐dependent manner (Huang *et al*., 2020). Nanodomains actively participate in plant signalling activities by concentrating their signalling molecules into the Lo phase on the PM (Duggan *et al*., 2008). Notably, upon pathogen infection, the assembly of nanodomains induces intermolecular and intramolecular interactions of membrane‐associated signalling proteins for modulating defence signalling transduction (Lv *et al*., 2017).

In this study, we found that a remorin homologue, REM1.2, was expressed mainly in the root epidermal, cortex and endodermal cells. In the same tissue, PM‐localised PIN2 modulated the asymmetric flux of auxin and root gravitropic growth. Exogenous SA application constrained the lateral movement of PIN2 molecules and condensed the diffusive PIN2 into protein clusters on the cell surface in a REM1.2‐dependent manner. As a result, SA‐induced PIN2 hyperclustering hampered auxin accumulation and impaired the root gravitropic response. Here, we propose that REM 1.2‐mediated PIN2 clustering is an alternative mechanism underlying the crosstalk between SA and auxin for the defence–growth trade‐off.

## Materials and Methods

### Plant growth and phenotype analysis

Seeds of *Arabidopsis thaliana* were sown on 0.8% agar containing half‐strength Murashige & Skoog medium (½MS) at 22°C under a 16 h : 8 h, light : dark photoperiod. Information on *rem1.2*, *rem1.2 1.3c* mutants, *XVE::REM1.2* and *GRF‐amiR* lines are given in a previous study (Huang *et al*., 2020). Except when specifically indicated, 4‐d‐old or 5‐d‐old seedlings were used for all experiments. DR5‐green fluorescent protein (GFP) or PIN2‐GFP on a *rem1.2* mutant background was generated by crossing DR5‐GFP or PIN2‐GFP plants with *rem1.2* mutants. DR5‐GFP or PIN2‐GFP on a *XVE::REM1.2* background was generated by *Agrobacterium* transformation of the XVE::REM1.2‐PMDC7B construct in DR5‐GFP or PIN2‐GFP plants.

### Used primers, vectors and cloning strategy

The primers used for genotyping, cloning and qRT‐PCR are listed in Supporting Information Table S1. Gateway vectors used for cloning and all the cloning strategies are listed in Table S2. For the generation of *pREM:GFP‐REM* constructs, 1.5 kb promoters of REMs with full‐length genomic DNA/cDNA of REMs were individually cloned into Gateway vectors using Gateway^®^ cloning technology (www.invitrogen.com) (Tables S1 and S2). The resultant constructs were introduced in *Columbia* (wild‐type) or individual *rem* mutants using the *Agrobacterium*‐mediated floral dip method.

### Root gravitropism assay

Seedlings were grown vertically for 4 d or 5 d, and any changes in the angle of the root tip beyond the direction of vertical growth were measured.

### Chemical treatment

For chemical preparation, a stock solution of SA was prepared using ethanol. Seedlings were treated with 100 µM SA (13 h or 24 h) for cell biology analysis. Methyl‐β‐cyclodextrin (Mβcd) was dissolved in ½MS medium at a final concentration of 2 mM for 15 h or 24 h treatment. 17β‐Estradiol (ES) was prepared in ethanol, and was further diluted to 5 µM in ½MS medium for the 13 h or 24 h pretreatment for cell biology analysis.

### Confocal microscopy observation

Seedlings were mounted on 0.8% agar ½MS chamber slides or liquid ½MS glass slides containing the indicated concentration of chemicals, then were immediately imaged. Images were taken using Zeiss LSM 880 (with Airyscan) or Leica SP8 confocal microscopes. The settings for excitation and detection were: GFP: 488 nm, 505–550 nm; red fluorescent protein (RFP): 554/561 nm, 565–650 nm. All images in a single experiment were captured using the same setting. The quantification method is described in the Supporting Information.

## Results

### SA regulation of root gravitropic growth involves remorin‐dependent membrane nanodomains

**Fig. 1:**
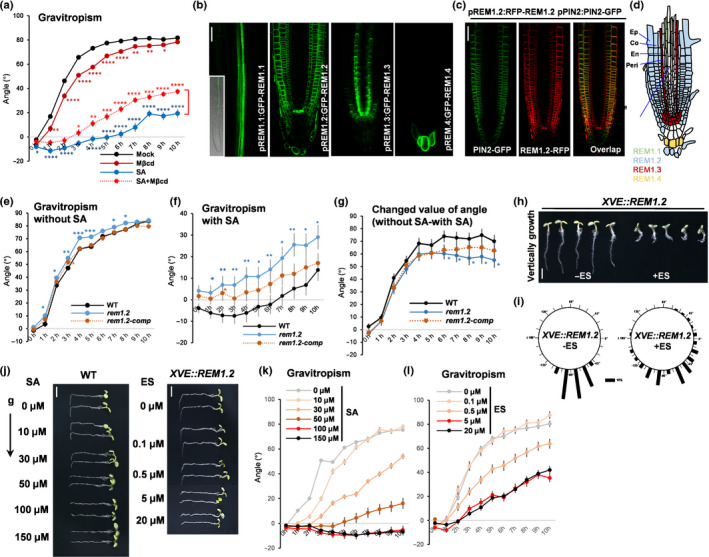
High salicylic acid (SA) and overexpressed REM1.2 impair root gravitropic responses in Arabidopsis. (a) Here, 4‐d‐old half‐strength Murashige and Skoog (½MS) medium grown wild‐type (WT) seedlings were transferred to methyl‐β‐cyclodextrin (Mβcd) (2 mM), SA (100 µM) or Mβcd plus SA‐containing medium for 10 h 90° reorientated growth, the deviated root tip angles along the vertical direction were measured (*n* > 50). Student’s *t*‐test was performed between the groups (mock vs SA, mock vs Mβcd, SA vs SA + Mβcd). (b–d) Here, 4‐d‐old transgenic plants of *pREM1.1:GFP‐REM1.1*, *pREM1.2:GFP‐REM1.2*, *pREM1.3:GFP‐REM1.3* and *pREM1.4:GFP‐REM1.4* showed distinct expression patterns in different root cell layers (b). Cell layer expression pattern of REM1.2 and PIN2 was visualised by red fluorescent protein (RFP) and green fluorescent protein (GFP) tagging, respectively (c). The cartoon with different colours (REM1.1 in green, REM1.2 in blue, REM1.3 in red, and REM1.4 in yellow) shows the representative expression pattern of these four REM1 members (d), and different cell layers were labelled (Ep, epidermis; Co, cortex; En, endodermis; Peri, pericycle; Ste, stele). (e–g) Here, 4‐d‐old ½MS‐grown WT, *rem1.2* and *rem1.2‐comp* seedlings were transferred to ½MS or 100 µM SA‐containing medium for 90° reorientation. The deviated angles and changed angles (comparing the angle difference of nontreated with SA‐treated seedlings) were tracked until 10 h. Student’s *t*‐test was performed between the groups (WT vs *rem1.2*, WT vs *rem1.2‐comp*). (h, i) *XVE::REM1.2* were germinated and continuously grown on 5 µM estradiol (+ES)‐supplemented ½MS medium for 5 d vertical growth (non‐estradiol‐containing medium (−ES) was used as control) (h). Root tip angles were measured as a percentage (compared with the length of 10%) (i) (*n* > 100). (j–l) Here, 4‐d‐old ½MS‐grown WT seedlings were transferred to ½MS or different concentrations of SA‐containing medium for 90° reorientation. Here, 4‐d‐old ½MS‐grown *XVE::REM1.2* seedlings were preincubated on medium different concentrations of estradiol for 13 h, and then reorientated for 90°. The deviated angles were tracked until 10 h. Bars: (b, c) 10 µm; (h, j) 1 mm. Error bars, SEM. *P*‐values were determined by two‐tailed Student’s *t*‐test assuming equal variances (*, *P* < 0.05; **, *P* < 0.01; ***, *P* < 0.001; ****, *P* < 0.0001).

We first examined whether elevated lipid packing in the Lo phase by SA would modulate a root gravitropic response. We transferred 4‐d‐old Arabidopsis wild‐type seedlings to SA‐containing medium and analysed the reorientated angles of roots after 90° reorientation. SA treatment resulted in a noticeable reduction in root gravitropism (Fig. 1a), which was consistent with previous reports (Philosoph‐Hadas *et al*., 2005; Du *et al*., 2013; Zhao *et al*., 2015; Pasternak *et al*., 2019; Tan *et al*., 2019). A dose‐dependent regulation of root agravitropic response was observed by serial concentrations of exogenous applied SA (Fig. 1j,k), suggesting a negative correlation between SA and gravitropism. Previous reported have shown that *npr1* remained sensitive to the stimulation of SA in gravitropism (Tan *et al*., 2019), which implied an NPR receptor‐independent mechanism for SA‐mediated root agravitropism. SA is known to increase the Lo of the PM in a remorin (REM) assembly‐dependent manner, but not the production of phytosterol and sphingolipid (Huang *et al*., 2020). We then examined Lo effects on SA‐suppressed gravitropism by utilising Mβcd to disassemble the nanodomain localised remorin proteins (Huang *et al*., 2020) and mimic the decrease in Lo (Jacobson *et al*., 2007). Mβcd, which has a central hydrophobic cavity able to form a 2 : 1 complex with cholesterol, is a well established pharmaceutical molecule for sequestrating cholesterol and is broadly used in mimicking lipid raft perturbation (Visco *et al*., 2014; Mahammad & Parmryd, 2015). Gravitropic angle analysis showed that Mβcd significantly attenuated the suppression of root gravitropic growth that was triggered by SA (Fig. 1a), suggesting a Lo‐dependent effect on root gravitropic response during SA signalling.

We next sought to test whether the key regulatory factor that underlines SA‐induced Lo, REM proteins, could directly mediate the crosstalk of SA and auxin signalling in regulating root gravitropism. REM is a protein family for nanodomain assembly on the cell surface that has 16 homologues in Arabidopsis with different assembly patterns and molecular distributions (Raffaele *et al*., 2009; Jarsch *et al*., 2014; Gronnier *et al*., 2017). We analysed the tissue‐specific expression of root‐abundant REM members, REM1.1–REM1.4, using transgenic Arabidopsis that expressed REM proteins tagged with GFP under the control of their native promoters (*pREM:GFP‐REM*). Surprisingly, REMs showed tissue‐specific expression patterns in the root tip, to some extent similar to PIN expression for the regulation of auxin transport (Feraru & Friml, 2008). *REM1.1* was mainly expressed along with the stele of the root maturation zone. *REM1.3* was expressed in the pericycle layer and the cells surrounding the quiescent centre (QC), whereas *REM1.4* was found only in the root cap cell of the tip region (Fig. 1b,d). Interestingly, *REM1.2* was expressed specifically in epidermis, cortex and endodermis cell layers of the root meristematic zone (Fig. 1b–d). Such a *REM1.2* expression pattern was reminiscent of the auxin transporter PIN2, which is expressed in the similar cell layers of epidermis and cortex in regulating auxin transport during the gravitropic response (Muller *et al*., 1998). Next, we asked whether REM1.2 is the downstream regulatory factor underlying the crosstalk of SA and auxin signalling. We examined the reorientated root tip angles of *rem1.2* mutant upon gravity stimulus in the presence or absence of exogenously supplemented SA. Without SA, *rem1.2* only showed a slightly earlier response to the gravity stimulus, compared with the wild‐type (Fig. 1e). However, the root gravitropism response to SA was desensitised significantly in *rem1.2* compared with the wild‐type (Fig. 1f,g). A complementation line *rem1.2‐comp* (*pREM1.2:GFP‐REM1.2* in *rem1.2* mutant, *rem1.2*‐complementation) (Huang *et al*., 2020) restored root gravitropic growth to SA supplementation similar to that of the wild‐type (Fig. 1e–g). The above results suggested that REM1.2 is highly involved in root gravitropism during SA signalling.

We then asked whether REM1.2 is the dominant remorin homologue for regulating root gravitropism. We additionally introduced the *REM1.3* mutation on the background of the *rem1.2* mutant. Compared with the *rem1.2* single mutant, the *rem1.2 1.3c* double mutant did not show obvious synergy in leading to the gravitropic phenotype, in the absence or presence of SA treatment (Fig. S1a–c). This finding suggests a predominant role of REM1.2 in mediating the crosstalk between SA and auxin. In addition, overexpression of REM1.2 in *XVE::REM1.2* under the control of the estradiol‐inducible promoter or constitutive overexpression of REM1.2 under the control of the 35S promoter (*35S:RFP‐REM1.2*), resulted in severely agravitropic roots, which not only phenocopied but exacerbated shortening of the primary root and multidirectional bending of the root tip as shown under SA treatment (Figs 1h,i, S1d,e,h). Short‐term induction of *XVE::REM1.2* by serial concentrations of estradiol displayed an inhibition of the gravitropic response in a dose‐dependent manner, which was consistent with the SA effect (Fig. 1j–l). The above pharmacological and genetic evidence supported an REM1.2‐dependent mechanism underlying SA‐triggered root agravitropism.

### SA impairs gravity‐induced asymmetric distribution of auxin and PIN2 in a REM1.2‐dependent manner

**Fig. 2:**
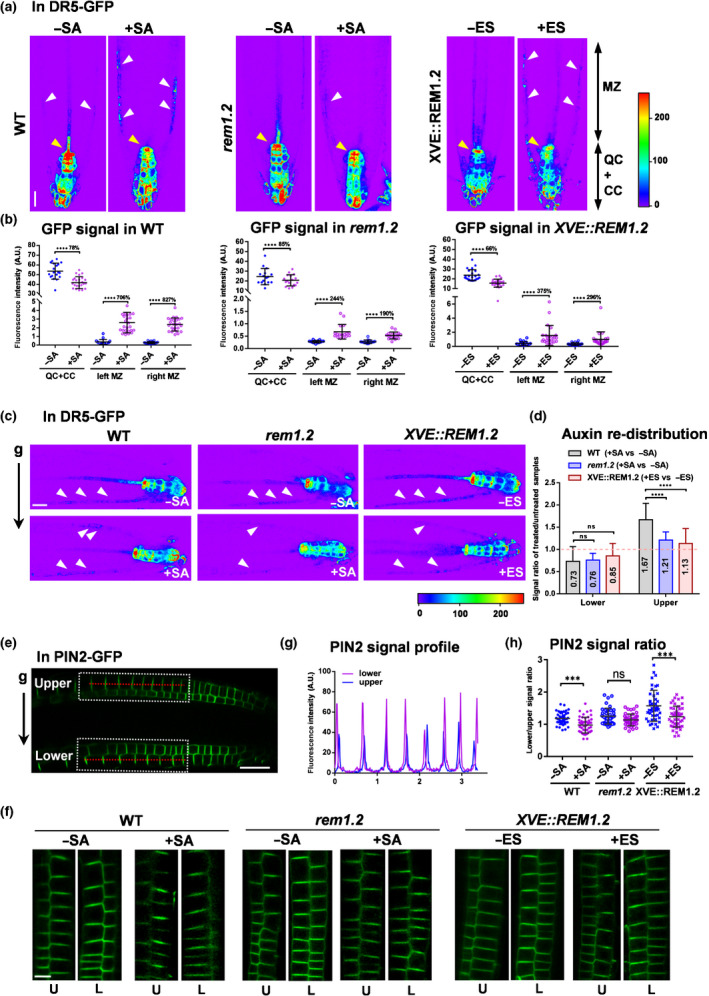
High SA and overexpressed REM1.2 impair asymmetric auxin and PIN2 redistribution during root gravitropic responses in Arabidopsis. (a, b) Here, 4‐d‐old DR5‐GFP in wild‐type (WT), *rem1.2* and *XVE::REM1.2* background seedlings were treated with 100 µM salicylic acid (SA) or 5 µM estradiol (ES) for 24 h, and GFP signal (fluorescence intensity (AU)) was measured in the quiescent centre (QC; yellow arrowheads) plus columella cell (CC) area of root tip and in the epidermis layer of root meristematic zone (MZ; white arrowheads), respective. Left and right indicate the left and right sides of the epidermis layer of root meristematic zone (b, from left to right, *n* = 19, 25, 19, 25, 19, 25 in WT; *n* = 15 in *rem1.2*; n = 22 in *XVE::REM1.2*). (c, d) Here, 4‐d‐old DR5‐GFP in WT and *rem1.2* background seedlings were treated with 100 µM SA for 13 h, and *XVE::REM1.2* DR5‐GFP seedlings were treated with 5 µM ES for 24 h. GFP signal profile in the upper and lower sides of each genotype was individually measured as white arrowheads indicated (c). The signal intensity of GFP was calculated individually in the upper and lower sides of roots. The columns showed the signal ratio of SA‐treated compared with untreated WT or *rem1.2*, and the signal ratio of estradiol‐induced compared with untreated *XVE::REM1.2* (fold change was indicated by the number) (d) (d, from left to right; *n* = 18, 17, 17, 18, 17, 17). (e–h) Here, 4‐d‐old PIN2‐GFP in WT and *rem1.2* background seedlings were treated with 100 µM SA for 13 h (untreated plants as controls), and *XVE::REM1.2* PIN2‐GFP seedlings were treated with 5 µM estradiol for 24 h. Here, 90°reorientation was performed for 4 h. GFP signal profile in the upper (U) and lower (L) sides of each genotype (e, white dots framed out) which was zoomed in (f) was individually measured in the epidermis layer of root meristematic zone along the red dot lines (e, g). The average signal ratio of lower/upper side was calculated in the chart (h, from left to right, *n* = 42, 47, 41, 44, 44, 47). Bars: (a, c, e) 10 µm; (f) and 5 µm (f). Error bars, SD. *P*‐values were determined by two‐tailed Student’s *t*‐test assuming equal variances (***, *P* < 0.001; ****, *P* < 0.0001; ns, not significant).

A gravitropic root response upon root tip reorientation is known to be a consequence of the asymmetric distribution of auxin (Band *et al*., 2012). We were next motivated to analyse the auxin level and its asymmetric distribution quantitatively in the root tip, with or without exogenous SA, using auxin response reporter DR5‐GFP (Ulmasov *et al*., 1997; Band *et al*., 2012). Auxin maximum was mainly distributed in the root tip region of wild‐type seedling around the QC and columella cell (CC) area (Fig. 2a). SA treatment resulted in a significant decrease in the DR5‐GFP signal at QC and CC regions, but there was an increase along the epidermis of the meristematic zone (MZ) in the wild‐type (Fig. 2a,b), indicating a disrupted auxin gradient during the SA response, which was consistent with recent findings (Pasternak *et al*., 2019; Tan *et al*., 2019). By contrast, SA‐caused ectopic auxin distribution was less significant in the *rem1.2* mutant (Fig. 2a,b), indicating a REM1.2‐dependent redistribution of auxin under SA signalling. In addition, DR5‐GFP distribution in root tip cells was also reduced by the high expression of REM1.2 in Arabidopsis that expressed *XVE::REM1.2* (Huang *et al*., 2020), in which an ectopically accumulated DR5‐GFP signal along the epidermis of the MZ was observed instead (Fig. 2a,b).

We further monitored auxin accumulation by comparing the asymmetrical signal of the DR5‐GFP reporter in the upper and lower layers of root after 90° reorientation. Without treatment, gravity caused more auxin deposition in the lower side of the root MZ region, compared with the upper side (Fig. 2c). Exogenous SA decreased auxin levels (0.73‐fold) significantly in the lower side of wild‐type roots, but increased auxin levels (1.67‐fold) in the upper side (Fig. 2c,d). However, SA impairment in the asymmetric distribution of auxin was markedly attenuated in the *rem1.2* mutant (Fig. 2c,d). In addition, high overexpression of REM1.2 could also impair the asymmetric auxin distribution upon gravity stimulus. Taken together, examination of auxin redistribution indicated that dynamic and functional assembly of REM1.2 is critical for regulating the SA‐induced asymmetric auxin deposition during the root gravitropic response.

The gravitropic response requires precise polar localisation of PIN2 to the shootward (apical) part in the root epidermis and rootward (basal) in root cortical cells (Muller *et al*., 1998), as well as an asymmetrical deposition at the upper side and lower side of the root tip in response to the gravity stimulus (Kleine‐Vehn *et al*., 2008). We next examined PIN2 redistribution in root after a 90° reorientation in wild‐type, *rem1.2,* and *XVE::REM1.2* seedlings, with or without SA treatment. PIN2 exhibited a higher signal intensity in the lower layer of cells than in the upper cells in the wild‐type (Fig. 2e–h), which was consistent with a previous report (Kleine‐Vehn *et al*., 2008). However, under both SA treatment and REM1.2 overexpression conditions, the gravity‐triggered redistribution of PIN2 was significantly compromised (Figs 2e–h, S1f,g). By contrast, *rem1.2* was less sensitive to SA treatment than the wild‐type by re‐depositing less PIN2 in the upper layers upon gravitropic stimulus (Fig. 2e–h). Taken together, the above results suggested that a tunable range of Lo in wild‐type cells probably provided flexible membrane tension for regulating fluid transportation of auxin, which is suppressed by SA through a substantial increase in Lo.

### SA stimulates PIN2 hyperclustering in a REM1.2‐dependent manner

**Fig. 3:**
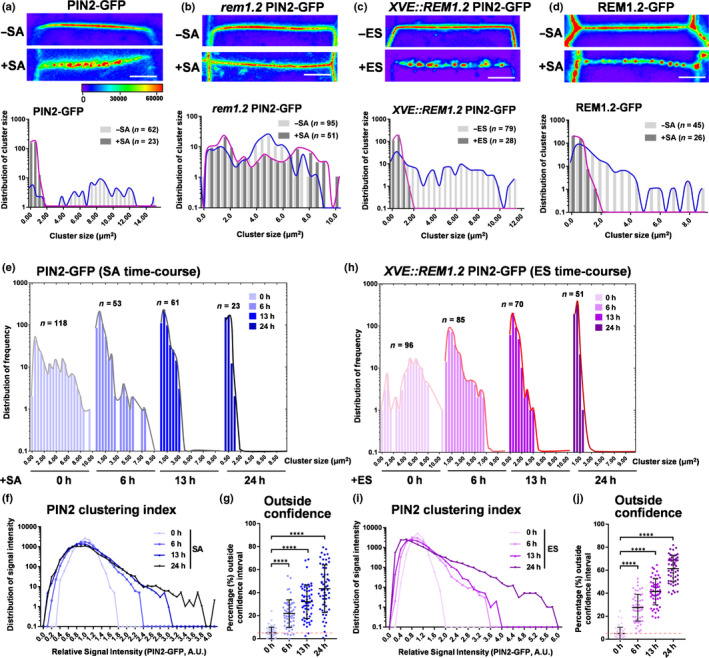
Salicylic acid (SA) promotes PIN2 clustering in a REM1.2‐dependent manner in Arabidopsis. (a–d) PIN2‐GFP in wild‐type (WT) and *rem1.2* background as well as *pREM1.2:GFP‐REM1.2* were treated with 100 µM SA for 24 h (nontreated plants were used as control) (a, b, d). *XVE::REM1.2* PIN2‐GFP seedlings were induced by 5 µM estradiol for 24 h (noninduced plants were the control) (c). Signal of REM1.2 and PIN2 was pseudocoloured. PIN2 and REM1.2 clusters were quantified according to their areas (cluster size in the x‐axis), and the distribution frequency of each cluster size was shown on the y‐axis. The cluster distributions with (+SA) or without salicylic acid (−SA) treatment were highlighted with purple and blue, respectively. (e–j) PIN2 clustering index and the percentage outside the 95% confidence of mock were quantified in PIN2‐GFP seedlings by 100 µM SA time‐course treatment (0, 6, 13 and 24 h) and in *XVE::REM1.2* PIN2‐GFP seedlings by 5 µM estradiol time‐course induction (0, 6, 13 and 24 h). Here, 0, 6, 13 and 24 h samples corresponded to: (e) *n* = 10 463 from 42 cells, 11 173 from 48 cells, 10 823 from 52 cells, 9025 from 37 cells in compartmentalisation index chart; (f) *n* = 11 437 from 44 cells, 12 295 from 55 cells, 11 571 from 55 cells, 12 683 from 56 cells in compartmentalisation index chart (AU, arbitrary unit). Pink dot lines marked the baseline of 5% (defined in the mock group) (g, j). Bars: (a–d) 1 µm. Error bars, SD. *P*‐values were determined by two‐tailed Student’s *t*‐test assuming equal variances (****, *P* < 0.0001).

Our previous study demonstrated that SA induced remorin clustering and enhanced PM compartmentalisation (Huang *et al*., 2020). To further understand the underlying mechanism of SA‐impaired asymmetric distribution of auxin and PIN2 during the gravitropic response, we next investigated the protein dynamics of PIN2 on the cell surface using subdiffraction‐limited Airyscan confocal microscopy (see Methods S1). We found that SA‐treated PIN2‐GFP displayed apparent discontinuity on the PM and compartmentalised into discontinued islands, and was heterogeneous in shape and size (Figs S2a, 3a). By contrast, PIN2 exhibited a relatively uniform distribution manner under other phytohormones‐supplemented conditions suggesting an SA‐specific response (Fig. S2d). The compartmentalisation index for PIN2 was then analysed quantitatively by grouping the PIN2‐GFP pattern into four categories depending on the cluster size, <0.2 µm^2^ (tiny island), 0.2–0.6 µm^2^ (small island), 0.6–1 µm^2^ (medium island), and >1 µm^2^ (long island) (Fig. S2c). In untreated wild‐type seedlings, 89% of PIN2‐GFP showed continuous distribution on the PM and were present as long islands. However, SA treatment markedly promoted the segmentation of PIN2‐GFP with enhanced interval distances between clusters (Figs 3a, S2a,c). According to the definition in a previous study of the PIN2 clustering pattern (Kleine‐Vehn *et al*., 2011) and recent findings (Li *et al*., 2020), we here found that SA‐triggered hyperclustering of PIN2 predominantly exhibited as tiny (31%) and small islands (61%) (Figs 3a, S2c). Interestingly, REM1.2 exhibited a similar hyperclustering pattern after SA treatment (Fig. 3d). We next tested PIN2 clustering patterns in the *rem1.2* mutant, in which a more attenuated hyperclustering of PIN2‐GFP was observed in response to SA (Fig. 3b). Strikingly, estradiol‐induced *XVE::REM1.2* also resulted in pronounced compartmentalisation of PIN2‐GFP on the PM (Fig. 3c). To simplify the quantification method, we compared the surface segmentation of PIN2 by measuring the discrete value, in which a more condensed PIN2 would result in a higher discrete value (termed the clustering index). Correspondingly, quantification of the PIN2 clustering index showed that SA triggered lateral redistribution of PIN2 into the heterogeneous clusters by showing a wider range of PIN2 signal intensity (Fig. S2e). We then analysed the significant differences between these groups by quantifying the percentage of PIN2 signal value beyond 95% confidence of the mock group (outside confidence) (Fig. S2b,f). With SA treatment, PIN2 proteins were compartmentalised into more condensed clusters in a time‐dependent manner (Fig. 3e–g). Consistently, time‐course induction of PIN2 hyperclustering also increased gradually, in a REM1.2 dose‐dependent manner, over the estradiol induction on *XVE::REM1.2* PIN2‐GFP seedlings (Figs S3a, 3h–j). We were next motivated to ask what was the functional correlation between the clustering level of PIN2 and auxin transportation under physiological conditions. Based on the dose‐dependent reduction of root gravity by SA (Fig. 1i,j), we further examined the PIN2‐GFP under the same conditions of SA treatments. We found that hyperclustering of PIN2 was gradually enhanced with increase in SA concentration (Fig. S3b,d–f). Consistently, a stepwise increase in REM1.2 expression by serial concentrations of estradiol also inhibited the gravitropic response and induced PIN2 hyperclustering in a dose‐dependent manner (Figs 1i,k, S3c,g–i). These above results indicated a negative correlation between gravitropism and PIN2‐GFP hyperclustering, which was in an SA‐dose‐dependent and REM‐expression level‐dependent manner. Such SA‐mediated PIN2 hyperclustering was also validated by direct detection via immunostaining using the PIN2 antibody (Fig. S2g–i). Therefore, the above data collectively demonstrated that surface clustering of PIN2 was enhanced by elevating either the dose of SA or REM1.2, which was expressed within the same tissue layers as PIN2.

Given that SA triggers remorin assembly for the nanoscale organisation of PM biomolecules (Huang *et al*., 2020) (Fig. 3d), we next sought to test whether remorin directly recruited PIN2 for surface nanoclustering. We immunostained *pREM1.2:GFP‐REM1.2* seedlings with GFP and PIN2 antibodies to compare the localisation of REM1.2 and PIN2 proteins, in the presence or absence of SA. In SA‐untreated samples, REM1.2 and PIN2 proteins localised uniformly on the PM, but colocalisation could not be resolved by confocal microscopy at the individual protein level (Fig. S4). Interestingly, the SA‐induced hyperclusters of PIN2 and REM1.2 did not exhibit apparent colocalisation (Fig. S4a–c). This finding suggests that the clustering of PIN2 proteins did not rely on direct interaction with REM1.2. PIN2‐clustering was triggered by the collaborative effects of the macromolecular assembly of REM1.2‐enhanced membrane compartmentalisation and the resulting increase in Lo.

### High‐order REM assembly suppresses CME

We next asked whether PIN2 hyperclustering was a consequence of the impairment of auxin maxima in the root. We perturbed the auxin biosynthesis pathway using l‐kynurenine (Kyn), a tryptophan (a precursor of auxin biosynthesis) analogue, as a competitive inhibitor of TAA1 (TRYPTOPHAN AMINOTRANSFERASE OF ARABIDOPSIS 1)/TARs in Arabidopsis (He *et al*., 2011), and 5‐(4‐chlorophenyl)‐4H‐1,2,4‐triazole‐3‐thiol (yucasin), which reduces YUCCA‐dependent auxin biosynthesis by inhibiting YUCCA (Nishimura *et al*., 2014). Furthermore, we enhanced the auxin concentration in the root tip by directly applying 1‐naphthaleneacetic acid (NAA). None of the seedling treatments with either Kyn alone, the mixture of Kyn and yucasin, or NAA could obviously affect clustering of PIN2 (Fig. S5a–d), suggesting that PIN2 hyperclustering did not derive from an increase or reduction in auxin levels.

**Fig. 4:**
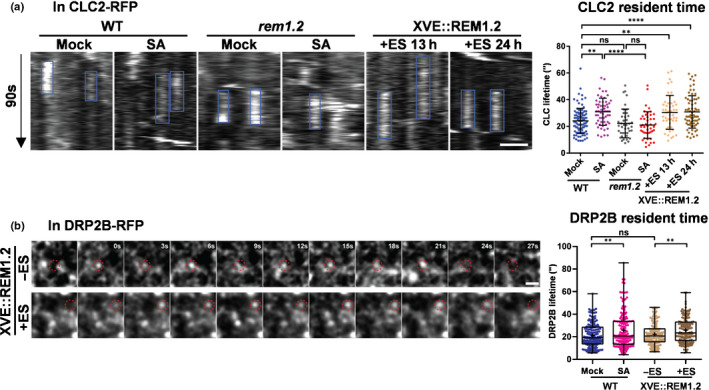
Compartmentalised nanodomains enforces the residence of PIN2 cargoes on the PM in Arabidopsis. (a) CLC2‐RFP lifetime on plasma membrane (PM) was quantitatively measured in wild‐type (WT), *rem1.2* and *XVE::REM1.2* root cells. Here, 4‐d‐old WT or *rem1.2* seedlings were treated with or without 100 µM salicylic acid (SA) for 24 h, and *XVE::REM1.2* seedlings were treated by 5 µM estradiol for 13 h or 24 h before imaging under variable‐angle total internal reflection fluorescence microscopy (VA‐TIRFM). The kymographs of the 90 s time course showed the CLC2‐RFP particle residents on the PM, which were indicated by blue boxes (a). Quantitatively analyses of CLC2‐RFP particle lifetimes were shown (*n* > 50 for each treatment). (b) The DRP2B‐RFP lifetime on PM was quantitatively measured in WT seedlings treated with or without 100 µM SA for 24 h, or in *XVE::REM1.2* seedlings treated by ethanol (control) or 5 µM estradiol for 24 h. Here, 4‐d‐old seedlings were used to do the treatments before imaging in root epidermal cells by VA‐TIRFM. Representative time‐course images showed typical DRP2B‐RFP particles (indicated by red dash circles) in *XVE::REM1.2* seedlings stayed on PM for 21 s (with ethanol treatment) and 24 s (with estradiol treatment). Quantitatively analyses of DRP2B‐RFP particle lifetimes in the indicated conditions described above were shown in the chart (*n* = 158, 162, 128, 138 from left to right). Bars: (a, b) 1 µm. Error bars, SD. *P*‐values were determined by two‐tailed Student’s *t*‐test assuming equal variances (**, *P* < 0.01; ****, *P* < 0.0001; ns, not significant).

Endocytic internalisation and recycling that are perpendicular to the PM coordinate lateral diffusion of surface molecules (including PIN2 cargoes) that jointly maintain the dynamic molecular distribution on the PM. Therefore, we were motivated to ask whether the known changes in PIN2 dynamics and SA were controlled by CME (Kitakura *et al*., 2011). We first examined the general CME process in SA‐treated or REM‐overexpressed seedlings. We generated stable transgenic plants that expressed clathrin‐light‐chain 2 (CLC2)‐RFP on *rem1.2* and *XVE::REM1.2* backgrounds, respectively. The lifetimes of CLC2 on the PM were analysed quantitatively by monitoring CLC2‐RFP in the root epidermis using variable‐angle total internal reflection fluorescence microscopy (VA‐TIRFM). Exogenous SA was applied to wild‐type seedlings, whereas estradiol was used to overproduce REM1.2 in *XVE::REM1.2*. Interestingly, both the above treatments resulted in significantly prolonged lifetimes of CLC2 on the PM (Fig. 4a), indicating a delayed CME. By contrast, the *rem1.2* mutant showed much less sensitivity to the SA effect, compared with the wild‐type, in changing the CLC2 lifetime (Fig. 4a). Similarly, the lifetime of another essential CME protein, dynamin‐related‐protein 2B (DRP2B)‐RFP (Fujimoto *et al*., 2010), was also prolonged by either SA treatment or REM1.2 overproduction, and also indicated a retarded CME (Fig. 4b). The above results demonstrated that CME was suppressed generally under increased levels of SA and in a REM1.2‐dependent manner.

### Compartmentalised nanodomains constrained the lateral diffusion of PIN2 on PM

The lipid properties and compartmentalisation of membranes impart distinct features for the distribution and interactions of membrane proteins (Owen *et al*., 2009; Bucciarelli *et al*., 2016; von Bulow *et al*., 2019). We next asked how the REM1.2‐mediated mechano‐properties of the PM regulated the lateral diffusion of PIN2‐GFP, and, thereby, the lateral clustering. We utilised a fluorescence recovery after photobleaching (FRAP) approach to evaluate the bulk and long‐distance mobility of PIN2 on the cell surface. We found that both SA treatment and REM1.2 overexpression in *XVE::REM1.2* seedlings caused significant retardation in PIN2 recovery (Fig. S6a,b). However, *rem1.2* exhibited reduced‐sensitivity to SA (14% upregulation of stable PIN2 fraction) by showing an enhanced recovery of PIN2‐GFP compared with the wild‐type seedlings (20% upregulation of stable PIN2 fraction) (Fig. S6a,b). To test whether SA‐mediated stabilisation of surface molecules was PIN2 specific, we performed a FRAP assay for a general PM marker GFP‐LT16B (low temperature induced, LTI) (Cutler *et al*., 2000) with or without exogenous SA (Fig. S6c,d). Interestingly, we also observed retardation of fluorescence recovery of GFP‐LT16B, suggesting a general reduction in biomolecular motility on the PM by SA treatment.

**Fig. 5:**
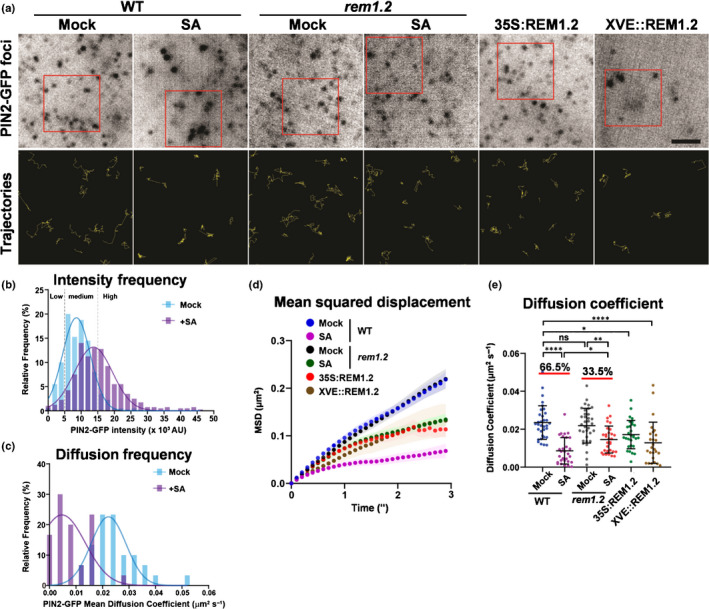
SA restricts PIN2 lateral diffusion in a REM1.2‐dependent manner in Arabidopsis. (a) PIN2‐GFP foci on the PM recorded by variable‐angle total internal reflection fluorescence microscopy (VA‐TIRFM) in 4‐d‐old wild‐type (WT) and *rem1.2* seedlings treated with or without 100 µM salicylic acid (SA) for 24 h, or in *35S:RFP‐REM1.2* and *XVE::REM1.2* (5 µM estradiol for 24 h). Root epidermal cells were observed. The lower panels showed 2× enlarged trajectories of the PIN2‐GFP particles in the red boxes during time‐lapse imaging for 6 s with 0.1 s interval. (b) Frequency distribution of PIN2‐GFP particle total intensity in WT seedlings treated without (Mock) or with 100 µM SA for 24 h as in (a). PIN2‐GFP particles were classified into three populations with high intensity (>15 × 10^3^ AU), medium intensity (5–15 × 10^3^ AU) and low intensity (<5 × 10^3^ AU), respectively. *n* = 400 particles randomly selected from more than three seedlings. (c) Frequency distribution of mean diffusion coefficient of PIN2‐GFP particles in WT seedlings that were treated without (Mock) or with 100 µM SA for 24 h. *n* = 30 region of interests (ROIs). (d, e) Quantitatively analysis of the PIN2‐GFP particle lateral dynamic on PM in indicated treatment conditions in (c). Mean square displacement (MSD) of PIN2‐GFP particle was plotted as a function of time at 0.1 s time resolution. Shaded areas indicated SE averaged from over 30 ROIs. For each ROI, the averaged MSD was further used to mean diffusion coefficient quantification (e) (*n* = 30 ROIs for each treatment). The percentages showed the comparison of SA‐treated to untreated samples. Bars, 2 µm (a). Error bars, SD. *P*‐values were determined by two‐tailed Student’s *t*‐test assuming equal variances (*, *P* < 0.05; **, *P* < 0.01; ****, *P* < 0.0001; ns, not significant).

We next investigated the molecular dynamics and clustering of PIN2 at the single molecular level to understand their initial assembly. We imaged cell surface‐associated PIN2‐GFP with a high signal‐to‐noise ratio by VA‐TIRFM with a spatial resolution at the nanoscale in studying PIN2 clustering at the single‐particle level. We analysed single‐molecule trajectories of PIN2‐GFP foci in wild‐type, *rem1.2* and *XVE::REM1.2*. With high‐imaging sensitivity, we observed the different populations of PIN2‐GFP punctates that were heterogeneous in intensity on the PM, suggesting the heterogeneity of PIN2 oligomerisation at the resting states (Fig. S7a). According to the signal intensity of PIN2 molecules, we divided these PIN2 populations into three groups (high, medium and low intensity) (Fig. S7b). Interestingly, the fluorescence intensity and lateral diffusion of PIN2‐GFP showed inverse correlations (Fig. S7c). Higher intensity particles show lower motility, and vice versa (Fig. S7a–c), which included the previously reported nonmobile population of PIN2 proteins (Kleine‐Vehn *et al*., 2011). We next quantified the frequency distribution of PIN2 signal intensity and diffusion coefficient with or without SA treatment. SA significantly elevated the frequency of high‐intensity PIN2‐GFP clusters and the immobile population (Fig. 5b,c). We next measured the overall mean squared displacement (MSD) and the diffusion coefficient of PIN2‐GFP particles. SA treatment significantly reduced the diffusion rate of PIN2 by showing a lower MSD and diffusion coefficient (Fig. 5d,e). Elevated REM1.2 produced more stabilised surface PIN2 molecules, which was in a dose‐dependent manner regarding the level of REM1.2 overproduction, in *35S:RFP‐REM1.2* or *XVE::REM1.2* (Fig. 5d,e). Interestingly, SA triggered a reduction in lateral motility of PIN2 in *rem1.2* by having a smaller decrease in diffusion coefficient (*c*. 33.5%), compared with the wild‐type (*c*. 66.5%) (Fig. 5e). These results demonstrated that the REM1.2 assembly tuned the lateral diffusion of surface PIN2 through regulating the mechanic properties of PM.

**Fig. 6:**
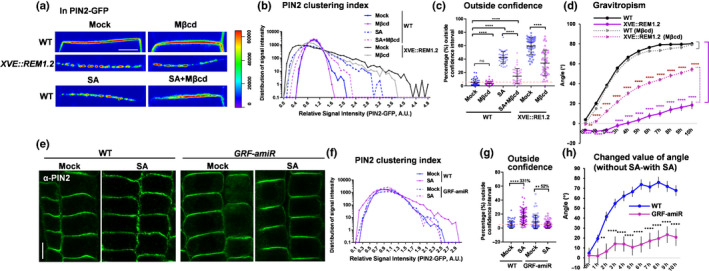
Plasma membrane compartmentalisation and lipid order regulate PIN2 clustering in Arabidopsis. (a–c) PIN2‐GFP in wild‐type (WT) and *XVE::REM1.2* background were treated with different chemicals: estradiol (ES) (5 µM) for 24 h, salicylic acid (SA) (100 µM) for 13 h, methyl‐β‐cyclodextrin (Mβcd) (2 mM) for 24 h, estradiol plus Mβcd or SA plus Mβcd co‐treatment (nontreated plants were used as control) (a). PIN2 clustering index and the percentage outside the 95% confidence of mock were measured in (b) and (c) (10 032 from 42 cells (WT–mock), 11 049 from 42 cells (WT‐Mβcd), 7649 from 42 cells (WT–SA), 10 750 from 45 cells (WT–SA–Mβcd), 10 443 from 51 cells (*XVE::REM1.2*‐mock), 10 673 from 43 cells (*XVE::REM1.2*‐Mβcd)). Pink dotted lines marked the baseline of 5% (defined in the mock group) (c). (d) Here, 4‐d‐old WT and *XVE::REM1.2* seedlings were continuously grown on ½MS medium, then transferred to 2 mM Mβcd, 5 µM estradiol, or 2 mM Mβcd plus 5 µM estradiol‐containing medium for 90° reorientation (10 h). Deviated root tip angles were measured (*n* > 50). (e–g) WT and *GRF‐amiR* were treated with 100 µM SA for 13 h (nontreated plants were used as control), and PIN2 distribution pattern was tested by immunostaining with PIN2 antibody (e). PIN2 clustering index and the percentage outside the 95% confidence of mock were measured in (f) and (g) (*n* = 11 257 from 49 cells (WT–mock), 11 501 from 58 cells (WT–SA), 15 207 from 65 cells (*GRF‐amiR*‐mock), 13 100 from 58 cells (*GRF‐amiR*‐SA)). Pink dot lines marked the baseline of 5% (defined in mock group) (g). Percentages showed the comparison of SA‐treated to untreated samples. (h) Here, 4‐d‐old seedlings of WT and *GRF‐amiR* line were continuously grown on ½MS medium with 5 µM estradiol or transferred to 5 µM estradiol plus 100 µM SA‐containing medium. They were subsequently reoriented by 90°. Root tip angles were measured according to the percentage of the deviated angle. SA sensitivity was quantified as the changed value of nontreated results compared with SA‐treated ones at each time point. Bars: (a) 1 µm; (e) 5 µm (e). Error bars, SD. *P*‐values were determined by two‐tailed Student’s *t*‐test assuming equal variances (**, *P* < 0.01; ****, *P* < 0.0001; ns, not significant).

We next tested whether the formation of PIN2 hyperclusters was derived from the retarded lateral diffusion of PIN2 molecules. We increased the lateral diffusion of PM‐associated proteins by reducing the surface Lo using Mβcd (Jones *et al*., 2010). Mβcd was applied to SA‐treated PIN2‐GFP seedlings, in which PIN2 displayed less hyperclustering after Mβcd co‐incubation, indicating the underlying roles of Lo and the constrained diffusion of PIN2 proteins for SA‐triggered PIN2 hyperclustering (Fig. 6a–c). In addition, estradiol‐induced REM1.2 expression triggered drastic lateral condensation of PIN2 proteins into discrete foci, phenocopying the SA‐stimulated condition (Fig. 6a–c). REM1.2 overexpression in *XVE::REM1.2* is known to induce the Lo drastically (Huang *et al*., 2020). Consistently, co‐incubation of Mβcd attenuated PIN2 hyperclustering significantly, which was previously induced by overexpressing REM1.2 (Fig. 6a–c). In addition, Mβcd also considerably prevented the agravitropic root phenotype of *XVE::REM1.2* (Fig. 6d), supporting the idea that higher Lo and retarded lateral diffusion of PIN2 led to PIN2 hyperclustering and thereby the consequences of root gravitropic responses.

We next examined how the assembly and packing of REM in nanodomains regulated PIN2 hyperclustering using the *GRF‐amiR* Arabidopsis line, which had silenced the remorin assembly factor, 14‐3‐3 proteins (Huang *et al*., 2020). By immunostaining experiments using the PIN2 antibody, *GRF‐amiR* plants were found to have a dramatic reduction in PIN2 hyperclustering that was previously triggered by SA in the wild‐type (Fig. 6e–g). Furthermore, *GRF‐amiR* seedlings displayed clearly impaired root gravitropism when they were grown on normal medium (Fig. S7d), and these phenotypes were consistent with the previously reported involvement of 14‐3‐3 members in the polar distribution of PIN proteins (Keicher *et al*., 2017). In addition, *GRF‐amiR* plants were much less responsive to SA‐triggered agravitropic responses than the wild‐type (Figs S7d,e, 6h). The above evidence strongly supported the idea that high‐order PM–nanodomain assembly by REMs constrained the lateral diffusion of PIN2 molecules that is critical for PIN2 hyperclustering during SA signalling.

## Discussion

**Fig. 7:**
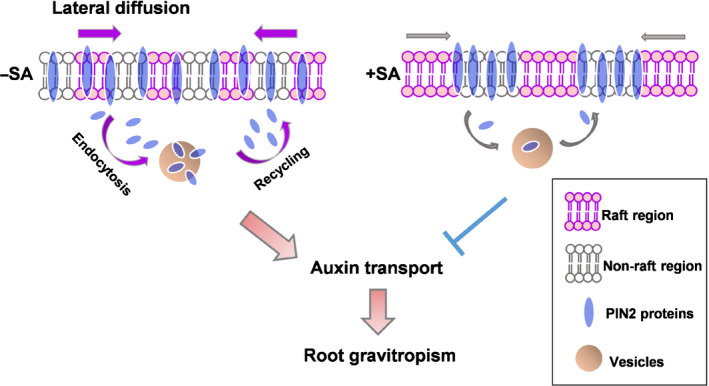
Speculative model of PIN2 clustering formation. The membrane system shows a highly packed liquid‐ordered phase (nanodomain region) and less packed liquid disordered phase (nonnanodomain region). Under low salicylic acid (SA) conditions (−SA), the ordered and disordered phases of lipids and PIN2 proteins are homogeneously distributed on the plasma membrane (PM). PIN2 proteins are trafficked among vesicles and the PM via dynamic endocytosis and lateral diffusion on the PM. High SA (+SA) causes nanodomain compartmentalisation and packing into a higher ordered lipid domain, in which endocytosis and lateral diffusive PIN2 movement are restricted. The constrained lateral movement of PIN2 leads to clustering.

Plants have evolved diverse mechanisms to regulate the chemical–physical properties of the PM and thereby fine tune signal transduction in response to different developmental and environmental stimuli. PIN protein regulates the directional flow of auxin that depends on its polarised PM localisation (Adamowski & Friml, 2015). During the defence response, plants accumulate high levels of SA that severely disrupt auxin accumulation and root gravitropism, indicating the antagonistic communication between SA and auxin signalling in the growth–defence trade‐off. Our previous study demonstrated that SA increased Lo and induced remorin clustering and compartmentalisation of PM nanodomains (Huang *et al*., 2020). Here, we found that SA crosstalk with auxin signalling that impaired auxin transport by hyperclustering PIN2‐GFP in a remorin‐dependent manner. Our quantitative analysis of PM‐localised PIN2 suggested that SA reduced the dynamics of different PM‐associated biomolecules by increasing PM order and nanodomain formation, such as the lateral diffusion and endocytic internalisation of PIN2 (Fig. 7). The counteractive effects in order changed remorin assembly by Mβcd or silenced remorin assembly factor 14‐3‐3 proteins, which greatly attenuated the SA effects on surface biomolecules. Compartmentalised PIN2 was evident as a suppressed asymmetrical transportation of auxin by impaired PIN2 redistribution upon gravity stimulus (Fig. 7). Our data provided insight into the regulatory mechanisms of PIN2‐dependent auxin transport in response to the high level of defence hormone SA, and might serve as an adaptive root strategy during plant growth response to defence.

### SA‐driven PM compartmentalisation regulates the dynamics of PM‐associated biomolecules

Fluid mosaic models and nanodomains of the PM describe its complex compartmentalisation structure in hosting the constrained and diffusive behaviour of surface molecules in motion (Singer & Nicolson, 1972; Malinsky *et al*., 2013). PM nanodomains provide platforms and scaffolds for the crosstalk between membrane‐associated proteins that are involved in signal transduction, immune responses, and the transportation of small molecules during environmental changes (Tapken & Murphy, 2015). For example, blue light irradiation stimulates the dimerisation of blue light receptor Phototropin1 (PHOT1) proteins, leading to the compartmentalisation of PHOT1, which is associated with nanodomains (Xue *et al*., 2018). Steroid hormone brassinosteroid (BR) promotes the partitioning of the BR receptor Brassinosteroid Insensitive 1 (BRI1) to membrane nanodomains that in turn stimulates BRI1 internalisation in response to BR (Wang *et al*., 2015). The modulation of the nanoclustering of receptor Flagellin Sensing 2 (FLS2) on the plant cell surface fine tunes the flagellum‐mediated innate immune responses under different conditions during plant–bacteria communication (Cui *et al*., 2018; Gronnier *et al*., 2020; Tran *et al*., 2020). Despite both immune receptor FLS2 and the growth receptor BRI1 forming nanoclusters on the cell surface, they distribute within distinct nanodomains, suggesting a spatial separation within the PM when responding to developmental or defence signals (Bucherl *et al*., 2017). The above studies suggested the notion that, in response to signalling molecules, PM nanodomains modulate the partitioning and oligomerisation of membrane‐associated signalling proteins to control signal transduction and developmental events. Here, we demonstrated that the SA‐induced increase of Lo restricted the dynamic behaviour of PIN2 on the PM and thereby impaired the root gravitropic response. This SA effect on membrane lipid composition was not PIN2‐specific and seemed to alter the behaviour of many membrane‐associated proteins, such as LT16B. How SA‐mediated membrane compartmentalisation regulates a diverse array of plant signalling molecules is worthy to unravel, especially the fundamental basis of crosstalk of complex signalling pathways by nanoscale molecular assembly and organisation.

### Nanodomain‐dependent PM compartmentalisation regulates PIN2 dynamics

The dynamic behaviour of PIN2 guarantees continuous flow and tunable auxin transportation. Both highly accumulated SA or assembly of REM1.2 on the cell surface impaired lateral motility and endocytic internalisation of PIN2, and led to the discontinued distribution of PIN2 into less mobile clusters. Hyperclustering of auxin efflux transporter PIN2 on the cell surface suppressed auxin accumulation and led to a low auxin level. Such surface PIN hyperclustering and PIN endocytosis are largely dependent on the level of nanodomain assembly and surface order. This is consistent with the known mechanisms by which SA increases REM oligomerisation, nanodomain assembly and Lo (Huang *et al*., 2020). A slowing down of endocytic internalisation of PIN could positively facilitate auxin flow by increasing the incidence of PINs at the cell surface, and which promotes auxin efflux in coordinating effective PIN recycling (Paciorek *et al*., 2005; Robert *et al*., 2010). However, under highly accumulated SA conditions, the reduced PIN2 dynamics, both laterally and perpendicular to the PM, was unable to coordinate such a positive feedback loop to support auxin flow. Instead of increasing auxin flux, SA impaired the internalisation and the lateral diffusion of PIN2, and thereby negatively regulated auxin efflux and disrupted asymmetrical auxin distribution in response to gravity stimulus. When exogenous SA treatment disrupted auxin maximum in the root tip, the PIN2 hyperclustering pattern was not influenced by the direct manipulation of auxin levels using synthesised auxin or an auxin biosynthesis inhibitor. Based on the previous conclusion that auxin inhibited PIN internalisation (Paciorek *et al*., 2005), it seems that the change in auxin level controls the general residence of PIN proteins on the PM, while the PIN clustering pattern is particularly affected by the organisation of membrane nanodomains.

The SA‐induced highly ordered lipid is likely to also impair CME by reducing the timely participation of the necessary endocytic constituents. Lo change by perturbing cholesterol levels is also known to modulate the invagination of clathrin‐coated pits and CME (Rodal *et al*., 1999). The detailed mechanisms by which REM assembly and Lo in plant cells tune CME are worthy of future investigations. It is conceivable that endocytic–exocytic dynamics needs to be coordinated with the appropriate lateral diffusion of PIN2 molecules to orchestrate the most effective auxin signalling upon demand.

### PIN2 hyperclustering and nanodomain assembly

The resting states of surface PIN2 showed heterogeneity at the low‐cluster states and lateral diffusion, and indicated the nature of the tunable dynamics of PIN2 in the molecular assembly and motility of PIN2 during auxin signalling. The striking inverse correlations between lateral motility and PIN2 clustering, and between PIN2 clustering and auxin transport, suggested a previously unknown mechanism in regulating PIN2 activities by altering protein motility and surface condensation (Kleine‐Vehn *et al*., 2011). Whether hyperclustering of PIN2 was derived from protein oligomerisation with self‐oligomerisation or protein condensation via multivalent interactions with binding partners is not yet known. SA induced the population of PIN2 particles to produce a bright signal and less mobile nature, implying that hyperclustering of PIN2 was similar to the oligomerisation of REM molecules upon SA stimulus (Huang *et al*., 2020). Future biophysical and cell biology studies, such as single‐molecule FRET or FRET between the same type of fluorophores (homoFRET) (Deng *et al*., 2014), would facilitate our understanding of the underpinning mechanisms.

Several auxin transportation‐relevant PM proteins, such as ABCB/multidrug resistance (ABCB) proteins (Borner *et al*., 2005; Titapiwatanakun *et al*., 2009; Demir *et al*., 2013; Yang *et al*., 2013), were also found to be nanodomain associated. ABCB protein is another type of auxin efflux transporter (Terasaka *et al*., 2005). Specific ABCB and PIN proteins form interactive pairs to enhance auxin transportation (Blakeslee *et al*., 2007). For example, PIN1 and ABCB19 co‐expression results in a higher rate of auxin transport than expression on their own. This synergistic effect is caused by enhanced PIN1 stability on the PM (Blakeslee *et al*., 2007). ABCB19 protein is anchored on the sterol/sphingolipid‐enriched membrane nanodomain fractions. Application of the membrane detergent Triton X‐100 significantly perturbed the interaction between PIN1 and ABCB19 within nanodomain fractions and disrupted the positioning of PIN1 on the PM (Titapiwatanakun *et al*., 2009). Another recent study found that functional PIN3 also formed PM nanoclusters (166.7 nm), which were distinct from the REM1.3 nanoclusters as they were bigger (231.0 nm) and more stable (McKenna *et al*., 2019). How these different signalling molecules on the surface are regulated regarding their diffusion and local condensation, and the details of their mechanisms are complex and mostly unclear. The tensegrity and mechanical scaffold of the cell wall‐plasma membrane‐cytoskeleton continuum could be highly involved in membrane protein diffusion and local condensation. For example, a cell wall perturbation by cellulose synthase inhibitor significantly increases the diffusion rate and cluster area of PIN3. It suggests that the cell wall and PM connections generate a traction force, resulting in a constrained movement and enlarged puncta size of PIN3. Consistently, cellulose‐based cell wall connections also restrict lateral diffusion of PIN1 cargoes and maintain PIN1 residence on the PM (Feraru *et al*., 2011). Furthermore the interconnected scaffolding system perpendicular to the PM surface, PM–nanodomain system also directly regulates the protein fluidity and, therefore, the compartmentalisation of the surface proteins in motion.

However, the molecular mechanisms by which the assembly level of nanodomains and surface Lo regulate the clustering and biochemical activities of the surface signalling molecules are extremely challenging to study. Genetic analysis of remorin remains limited due to the functional redundancy of 16 remorin homologues in Arabidopsis. Currently, we still have limited knowledge in the macromolecular assembly of remorins on the PM in the nanoscale as well as the multifaceted biological functions. Here, we focused on the remorin 1 subfamily, including REM1.1, 1.2, 1.3 and 1.4 four members. These four REMs showed tissue‐specific expression patterns in the root tip, in which REM1.2 is specifically expressed in a PIN2‐similar root cell layer. However, we cannot exclude the potential participation of other remorin members in regulating root gravitropic response. Nevertheless, we showed that REM1.2 is one of the critical underlying regulators that serve as the gatekeeper to control the threshold for the exponential growth of nanoclustering, typical biophysical phenomena for nanoclustering and phase‐separation mediated nanocondensation of biomolecules, including lipids and proteins (Case *et al*., 2019; Platre *et al*., 2019; Xie *et al*., 2019; Choi *et al*., 2020; Jaillais & Ott, 2020; Narita, 2020). The preexisting REM1.2 clusters balanced PM compartmentalisation under physiological conditions, which seem to be well coordinated with surface PIN2 at a low level of molecular clustering. However, upon SA stimulation, REM1.2 exhibited a critical role in modulating the amplification of PIN2 preparation for clustering. Therefore, remorin might display differential regulatory functions in controlling surface molecular‐mediated signalling transduction under different environmental conditions with other mechanical or chemical cues.

For auxin‐involved plant morphogenesis, the surface nanodomain structure was found to be crucial in maintaining the stability of membrane‐associated auxin transporters and enhanced their activity (Titapiwatanakun *et al*., 2009). However, under SA high‐accumulated conditions and REM overexpression, PIN underwent complex assembly into heterogeneous condensed and immobilised foci, within a wide range of sizes up to several micrometres. As a result, auxin transportation and signalling were largely compromised, this suggested a dynamic range of PIN assembly on the cell surface in tuning its activity by either elevating or attenuating the activities of auxin transport and plant development. Future studies should also identify whether PIN2 hyperclustering is derived from PIN2–PIN2 self‐interaction or synergised with other binding partners with multivalent interactions. The tunable control of the clustering and spatial confinement of PIN2, in a spatial–temporal controlled manner, exhibited complex regulatory mechanisms for the plant to either maintain, activate or suppress auxin transport, in response to pathogenic infection, in balancing the defence–growth trade‐off.

## Author contributions

MK, ZM, YM and XC designed research; MK, ZM, DW, YS, CW, DH, ZC performed the research; LY contributed new analytic tools; ST, RL and JF helped the manuscript interpretation and analysed data; and YM and XC wrote the paper. MK and ZM contributed equally as co‐first authors, and YM and XC contributed equally as co‐corresponding authors.

## Supporting information


**Fig. S1** REM1.2‐mediated Arabidopsis root gravitropic responses during SA signalling.
**Fig. S2** Surface PIN2‐GFP clustering by SA treatment and REM1.2 overexpression in Arabidopsis.
**Fig. S3** Enhanced surface clustering of PIN2 by dose increasing SA or REM1.2 expression in Arabidopsis.
**Fig. S4** Surface clustering of Arabidopsis PIN2 and REM1.2 are not co‐localised.
**Fig. S5** Auxin level does not influence PIN2 clustering in Arabidopsis.
**Fig. S6** SA restricts lateral diffusion of membrane‐associated proteins in Arabidopsis.
**Fig. S7** Arabidopsis PIN2‐GFP clusters and dynamics on plasma membrane.
**Methods S1** Quantification method.
**Table S1** List of primers used for genotyping and qRT‐PCR analysis.
**Table S2** Cloning strategy.
Please note: Wiley Blackwell are not responsible for the content or functionality of any Supporting Information supplied by the authors. Any queries (other than missing material) should be directed to the *New Phytologist* Central Office.
