# Supplementary material for: Salicylic acid regulates PIN2 auxin transporter hyperclustering and root gravitropic growth via Remorin‐dependent lipid nanodomain organisation in Arabidopsis thaliana
Source: New Phytol. 2020 Sep 30;229(2):963–78. doi: 10.1111/nph.16915 (PMC7821329; doi:10.1111/nph.16915)
Supplement: Supplementary file 1 — Fig. S1 REM1.2‐mediated Arabidopsis root gravitropic responses during SA signalling. Fig. S2 Surface PIN2‐GFP clustering by SA treatment and REM1.2 overexpression in Arabidopsis. Fig. S3 Enhanced surface clustering of PIN2 by dose increasing SA or REM1.2 expression in Arabidopsis. Fig. S4 Surface clustering of Arabidopsis PIN2 and REM1.2 are not co‐localised. Fig. S5 Auxin level does not influence PIN2 clustering in Arabidopsis. Fig. S6 SA restricts lateral diffusion of membrane‐associated proteins in Arabidopsis. Fig. S7 Arabidopsis PIN2‐GFP clusters and dynamics on plasma membrane. Methods S1 Quantification method. Table S1 List of primers used for genotyping and qRT‐PCR analysis. Table S2 Cloning strategy. Please note: Wiley Blackwell are not responsible for the content or functionality of any Supporting Information supplied by the authors. Any queries (other than missing material) should be directed to the New Phytologist Central Office. [file NPH-229-963-s001.pdf]

New Phytologist

New Phytologist Supporting Information

Article title: Salicylic Acid regulates PIN2 auxin transporter hyper-clustering and root gravitropic growth via Remorin-dependent lipid nanodomain organization
Authors: Meiyu Ke, Zhiming Ma, Deyan Wang, Yanbiao Sun, Chenjin Wen, Dingquan Huang, Zichen Chen, Liang Yang, Shutang Tan, Ruixi Li, Jiří Friml, Yansong Miao and Xu Chen
Article acceptance date: 25 August 2020

The following Supporting Information is available for this article:

Fig. S1 REM1.2-mediated *Arabisopsis* root gravitropic responses during SA signaling.

Fig. S2 Surface PIN2-GFP clustering by SA treatment and REM1.2 overexpression in *Arabidopsis*.

Fig. S3 Enhanced surface clustering of PIN2 by dose increasing SA or REM1.2 expression in *Arabidopsis*.

Fig. S4 Surface clustering of *Arabidopsis* PIN2 and REM1.2 are not co-localized.

Fig. S5 Auxin level does not influence PIN2 clustering in *Arabidopsis*.

Fig. S6 SA restricts lateral diffusion of membrane-associated proteins in *Arabidopsis*.

Fig. S7 *Arabidopsis* PIN2-GFP clusters and dynamics on plasma membrane.

Table S1 List of primers used for genotyping and qRT-PCR analysis

Table S2 Cloning Strategy

Methods S1 Quantification method

Fig. S1 REM1.2-mediated *Arabisopsis* root gravitropic responses during SA signaling.

(A-C) 4-day-old WT, *rem1.2* and *rem1.2 1.3c* seedlings were continuously grown on 1/2 MS (A) or transferred to 100 μM salicylic acid (SA)-containing medium (B) for 90° reorientation (10h). The deviated root tip angles were measured (n>45) and the changed values of angle were counted in C.

(D) Root phenotype of 5-day-old WT and *35S:RFP-REM1.2* seedlings.

(E) REM1.2 transcript was examined by qRT-PCR in *pREM1.2:RFP-REM1.2*, *35S:RFP-REM1.2* and *XVE::REM1.2* seedlings (with PIN2-GFP).

(F-G) 4-day-old PIN2-GFP in WT and *35S:RFP-REM1.2* background seedlings were performed 90° reorientation for 4h. GFP signal profile in the upper and lower sides of each genotype was individually measured. The average signal ratio of lower/upper surfaces was calculated in the chart (G, from left to right, n=13 and 14 seedlings).

(H) 4-day-old 1/2 MS-grown WT, *XVE::NHL19* (NDR1/HIN1-like 19) seedlings were transferred to 5 μM estradiol-containing medium for a 10 h pretreatment, and then reorientating 90° . The deviated angles were tracked till 10h. *XVE::NHL19* was used as a control (expressing another unrelated protein) of *XVE::REM1.2* for analysis of gravitropic phenotype.

Scale bars, 1 mm (D) and 5 μm (F). Error bar= S.E.M in A-C, H; S. D. in E, G. P-values were determined by two-tailed Student's t-test assuming equal variances (*p < 0.05, **p < 0.01, ***p< 0.001, ****p< 0.0001)(each t-test analysis was performed between the group between WT and *rem* mutants, these were no significant differences between *rem1.2* and *rem1.2 1.3* groups in A-C).

Supp. Fig. 1

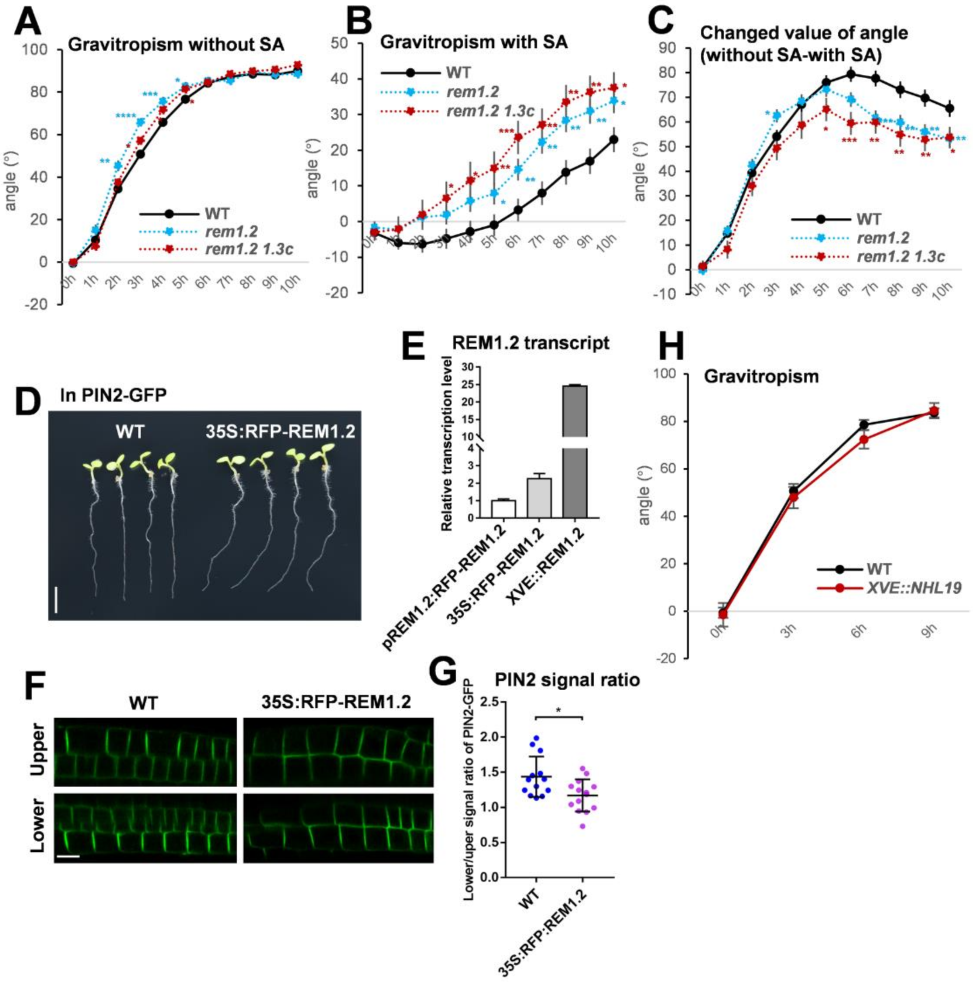

A
Gravitropism without SA
angle (°)
WT
rem1.2
rem1.2 1.3c
B
Gravitropism with SA
C
Changed value of angle (without SA-with SA)
D
In PIN2-GFP
WT
35S:RFP-REM1.2
E
REM1.2 transcript
Relative transcription level
pREM1.2:RFP-REM1.2
35S:RFP-REM1.2
XVE::REM1.2
H
Gravitropism
XVE::NHL19
F
Upper
Lower
G
PIN2 signal ratio
Lower/uper signal ratio of PIN2-GFP
35S:RFP:REM1.2

Fig. S2 Surface PIN2-GFP clustering by SA treatment and REM1.2 overexpression in *Arabidopsis*.

(A) Representative pictures showed the two typical PIN2 distribution patterns with non-cluster one and cluster-abundant one. Dot lines and red arrows highlight the cluster pattern and interval length.

(B) Representative pictures showed the quantification method of percentage outside 95% confidence.

(C) PIN2 and REM1.2 clusters were divided into four categories according to their areas, and the distribution frequency (%) was measured, respectively.

(D-F) PIN2-GFP seedlings were treated with different phytohormones (100 μM salicylic acid (SA) 13h, 1 μM abscisic acid (ABA) 2h, 1 μM 24-epibrassinolide (eBL) 6h, 50 μM gibberellin (GA_3, an active form of GA) 4h, 100 μM methyl jasmonate (MeJA) 1h). PIN2 clustering index and the percentage outside 95% confidence of mock were quantified. Pink dot lines marked the baseline of 5% (defined in the mock group) (F).

(G-I) WT seedlings were treated with 100 μM SA for 13 h (non-treated plants were used as control), and PIN2 distribution pattern was tested by immunostaining with PIN2 antibody (G). PIN2 clustering index and the percentage outside 95% confidence of mock were measured in H and I (n=12534 from 64 cells (-SA), 17052 from 80 cells (+SA)). The images in the bottom displayed the 2× enlarged views of boxed areas in the original images (G). Pink dot lines marked the baseline of 5% (defined in mock group) (I).

Scale bars, 1 μm (A, D) and 5 μm (G). Error bar=S.D (C, F, I). P-values were determined by two-tailed Student's t-test assuming equal variances (**p < 0.01, ****p< 0.0001, ns, not significant).

Supp. Fig.2

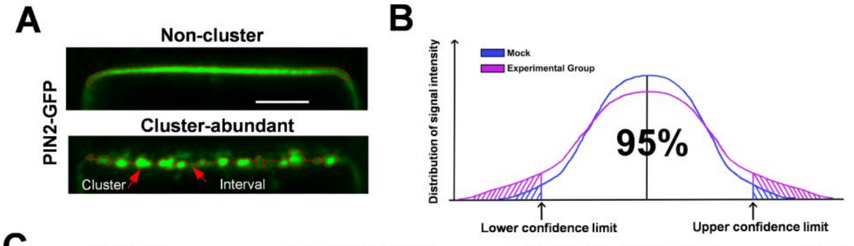

C

PIN2-GFP			*rem1.2* PIN2-GFP			*XVE::REM1.2* PIN2-GFP			REM1.2-GFP		
area (μm²)	-SA	+SA	area (μm²)	-SA	+SA	area (μm²)	-ES	+ES	area (μm²)	-SA	+SA
0-0.2	2.56±2.33%	31.02±4.63%	0-0.2	1.76±1.53%	0.00±0.00%	0-0.2	5.68±6.12%	20.69±7.83%	0-0.2	2.75±1.28%	38.50±8.95%
0.2-0.6	7.01±4.76%	61.36±7.52%	0.2-0.6	3.18±2.77%	6.81±0.82%	0.2-0.6	19.40±9.09%	68.17±2.32%	0.2-0.6	28.74±8.40%	54.89±5.12%
0.6-1	1.04±1.80%	5.88±0.81%	0.6-1	6.70±2.90%	6.05±3.58%	0.6-1	9.07±1.75%	10.49±9.14%	0.6-1	19.93±5.50%	6.32±4.95%
>1	89.39±7.49%	1.74±2.34%	>1	88.36±5.09%	86.13±2.45%	>1	64.95±12.67%	0.66±1.13%	>1	48.58±9.34%	0.28±0.49%

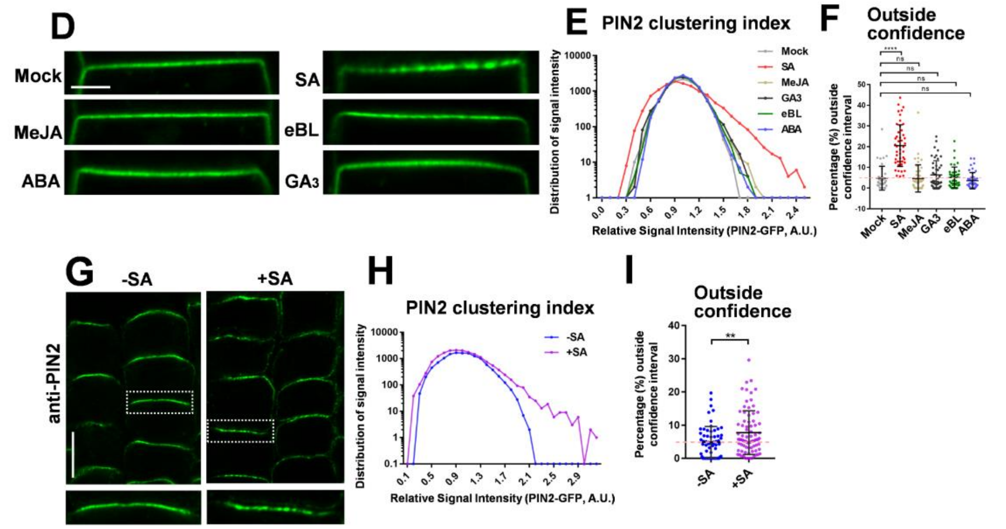

Fig. S3 Enhanced surface clustering of PIN2 by dose increasing SA or REM1.2 expression in *Arabidopsis*.

(A) PIN2-GFP seedlings were treated by 100 µM salicylic acid (SA) (0, 6, 13, and 24 h) and *XVE::REM1.2* PIN2-GFP seedlings were induced by 5 µM estradiol (ES) (0, 6, 13, and 24 h). Corresponding to the quantification in Fig. 3E-J.

(B-I) PIN2-GFP seedlings were treated by dose-concentration of SA (0, 10, 30, 50, 100 and 150 µM) and *XVE::REM1.2* PIN2-GFP seedlings were induced by dose-concentration of estradiol (0, 0.1, 0.5, 5 and 20 µM). PIN2 clustering index and the percentage outside 95% confidence of mock were quantified (D, E: 0, 10, 30, 50, 100 and 150 µM SA corresponding to n=8486 from 31 cells, 8530 from 33 cells, 8319 from 33 cells, 8285 from 35 cells, 8372 from 45 cells, 8338 from 40 cells; G, H: 0, 0.1, 0.5, 5 and 20 µM estoadiol corresponding to n=10188 from 43 cells, 10100 from 44 cells, 10125 from 45 cells, 9873 from 47 cells, 9824 from 50 cells). The correlation between reorientated angle at 10h and PIN2 clustering index (outside 95% confidence) were generated in F and I charts.

Scale bars, 1 µm. Error bar=S.D. P-values were determined by two-tailed Student’s t-test assuming equal variances (****$p < 0.0001$, ns, not significant).

Supp. Fig.3

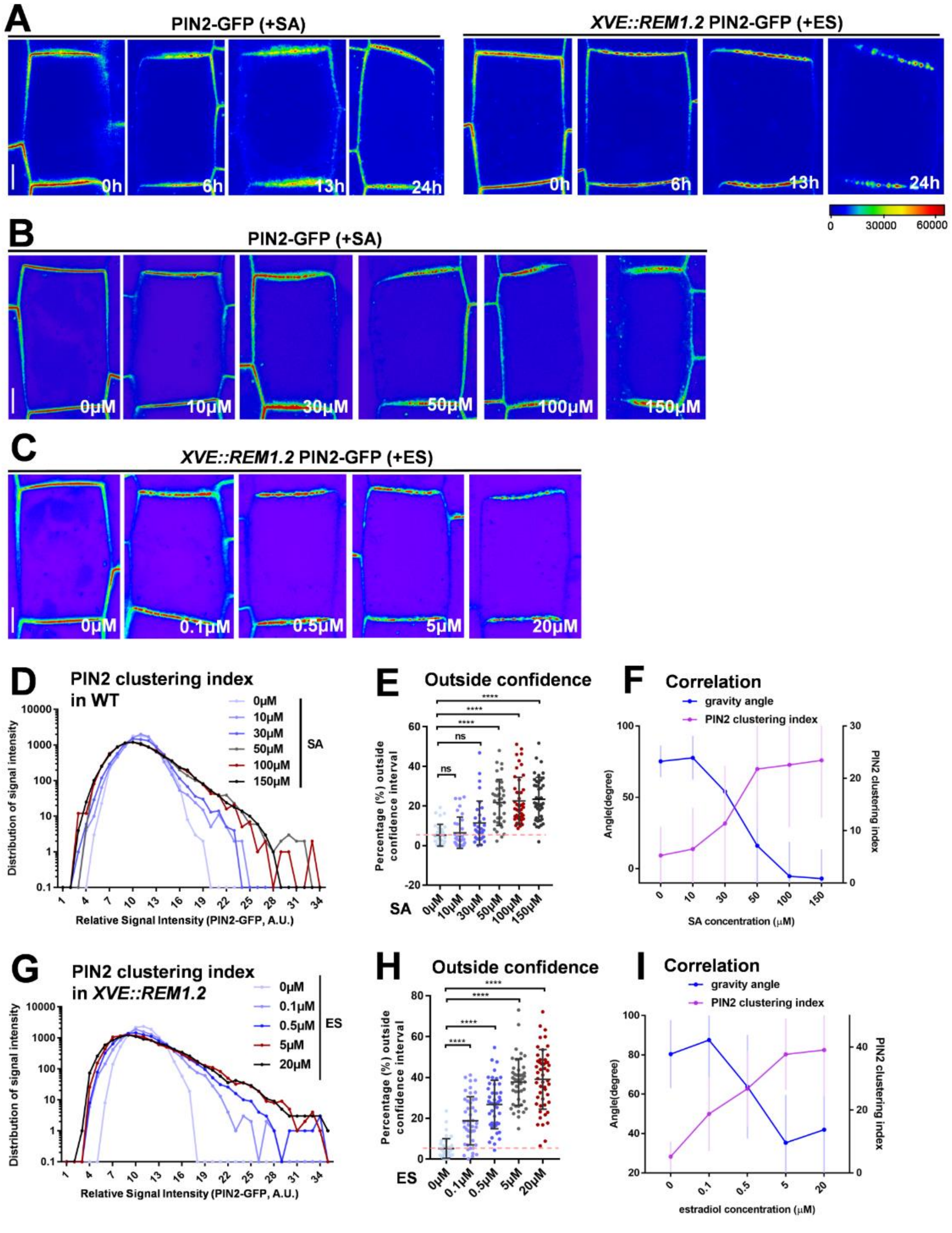

A
PIN2-GFP (+SA)
XVE::REM1.2 PIN2-GFP (+ES)
0h
6h
13h
24h
0
30000
60000
B
PIN2-GFP (+SA)
0μM
10μM
30μM
50μM
100μM
150μM
C
XVE::REM1.2 PIN2-GFP (+ES)
0μM
0.1μM
0.5μM
5μM
20μM
D
PIN2 clustering index in WT
Distribution of signal intensity
Relative Signal Intensity (PIN2-GFP, A.U.)
SA
E
Outside confidence
Percentage (%) outside confidence interval
ns
****
F
Correlation
gravity angle
PIN2 clustering index
Angle(degree)
SA concentration (μM)
G
PIN2 clustering index in XVE::REM1.2
ES
H
Outside confidence
I
Correlation
estradiol concentration (μM)

Fig. S4 Surface clustering of *Arabidopsis* PIN2 and REM1.2 are not co-localized.

(A-C) Co-localization of REM1.2 and PIN2 proteins was tested by immunostaining in *pREM1.2:GFP-REM1.2* seedlings (with or without 100 μM salicylic acid (SA) treatment) by GFP (red signal) and PIN2 (green signal) antibodies. The below panels were shown 5× enlarged views of boxed areas in the original images (B). Co-localization was evaluated using the Pearson correlation coefficient by randomly selected region of interests (ROIs) on PM (n=43) (C).

Scale bars, 5 μm. Error bar=S.D (C).

Supp. Fig. 4

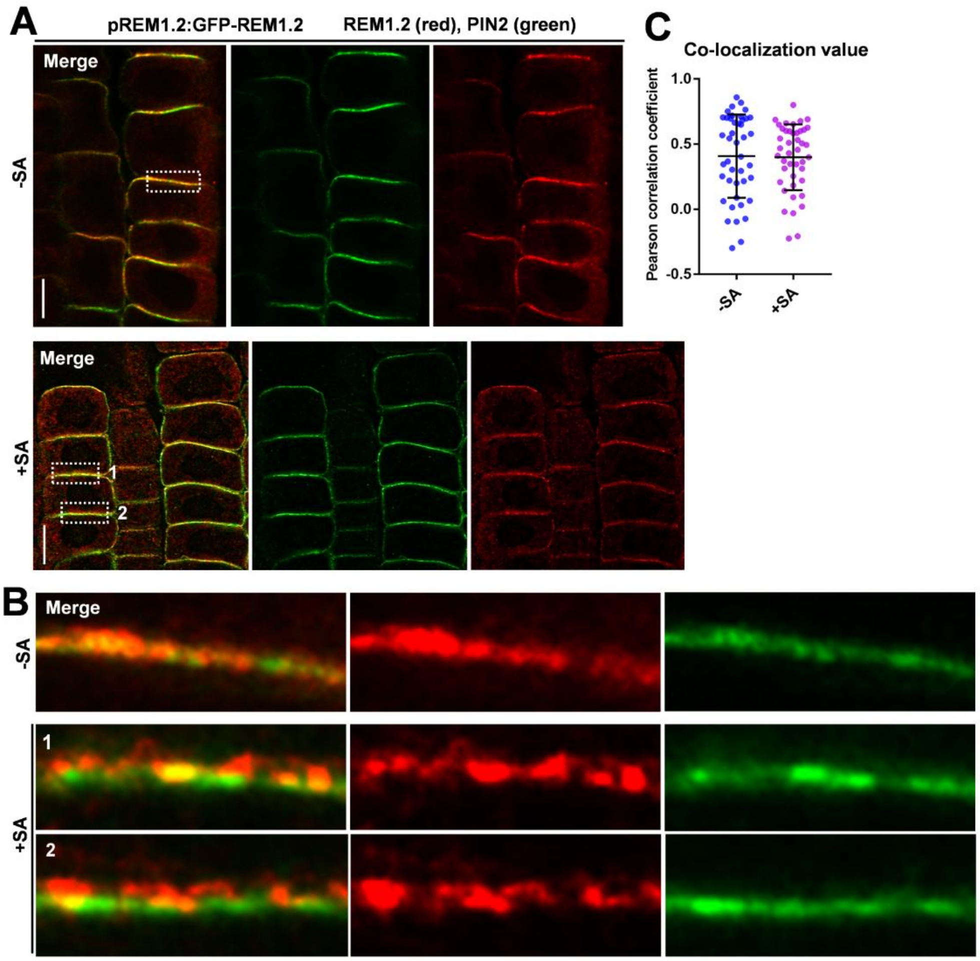

A
pREM1.2:GFP-REM1.2
REM1.2 (red), PIN2 (green)
Merge
-SA
+SA
1
2
B
C
Co-localization value
Pearson correlation coefficient
1.0
0.5
0.0
-0.5
-SA
+SA

Fig. S5 Auxin level does not influence PIN2 clustering *in Arabidopsis*.

(A-D) DR5-GFP and PIN2-GFP seedlings were treated by naphthalene acetic acid (NAA) (100 nM), salicylic acid (SA) (100 μM), L-Kynurenine (Kyn) (50 μM), Kyn (30 μM) puls 5-(4-chlorophenyl)-4H-1,2,4-triazole-3-thiol (yucasin) (20 μM) for 13h. GFP signal was measured in the quiescent center (QC) and columella cell (CC) areas (B) (from left to right: n=26, 22, 19, 16 and 15). PIN2 clustering index and the percentage outside 95% confidence of mock were quantified (from left to right: n=8215 from 32 cells, 8290 from 36 cells, 8267 from 37 cells, 8350 from 38 cells, 8204 from 36 cells) (C-D).

Scale bars, 5 μm (A) and 1 μm (C). Error bar=S.D. P-values were determined by two-tailed Student's t-test assuming equal variances (**$p < 0.01$, ****$p < 0.0001$, ns, not significant).

Supp. Fig.5

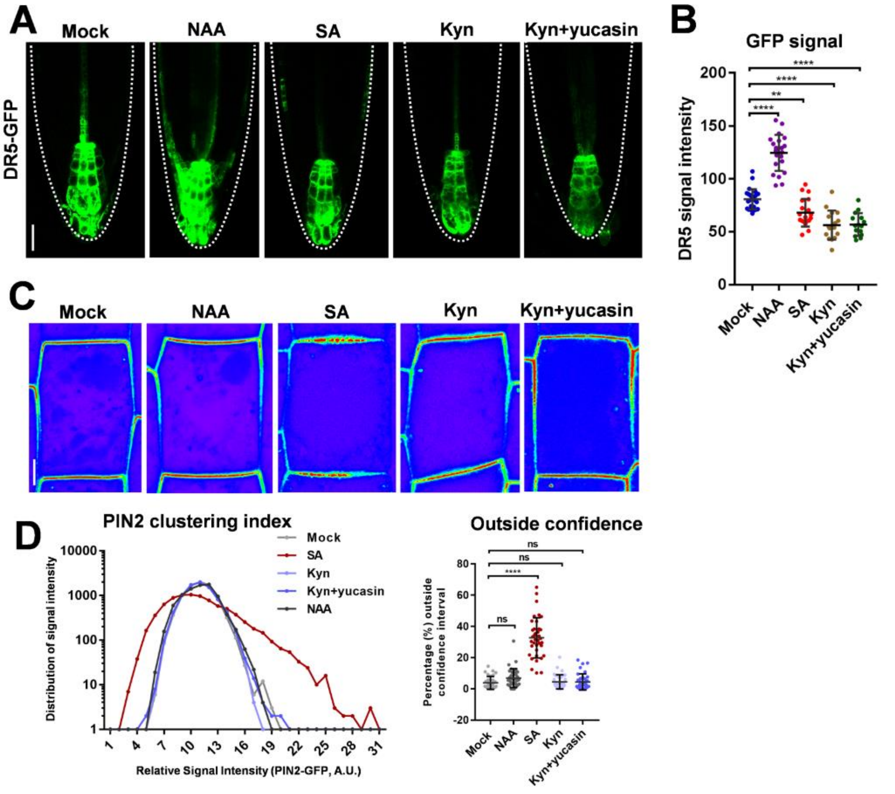

A
Mock
NAA
SA
Kyn
Kyn+yucasin
DR5-GFP
B
GFP signal
DR5 signal intensity
****
**
Mock
NAA
SA
Kyn
Kyn+yucasin
C
Mock
NAA
SA
Kyn
Kyn+yucasin
D
PIN2 clustering index
Distribution of signal intensity
Relative Signal Intensity (PIN2-GFP, A.U.)
Mock
SA
Kyn
Kyn+yucasin
NAA
Outside confidence
Percentage (%) outside confidence interval
ns
****

Fig. S6 SA restricts lateral diffusion of membrane-associated proteins *in Arabidopsis*.

(A-B) PIN2-GFP in WT and *rem1.2* seedlings (with or without 100 μM salicylic acid (SA) for 13 h pretreatment), *XVE::REM1.2* PIN2-GFP (with 5 μM estradiol (ES) for 24h pre-incubation) were photo-bleached (A). The recovery and stable fraction of PIN2 signals was measured based on the PIN-GFP in (A) (from left to right, n=10, 9, 12, 13, 10, 9 seedlings) (B). The percentages in the B chart indicate the increased percentages of treated compared with untreated samples.

(C-D) SA-treated LT16B-GFP (with or without 100 μM SA for 13h pretreatment) were photo-bleached. The recovery efficiency of LT16B signal was measured as the curves shown and the stable LI16B fraction was quantified (D, from left to right, n=15 and 24). The percentages in the D chart indicate the increased percentages of treated compared with untreated samples.

Scale bars, 1 μm (A, C). Error bar=S.D. P-values were determined by two-tailed Student's t-test assuming equal variances (*p<0.05, ***p< 0.001, ns, not significant).

Supp. Fig. 6

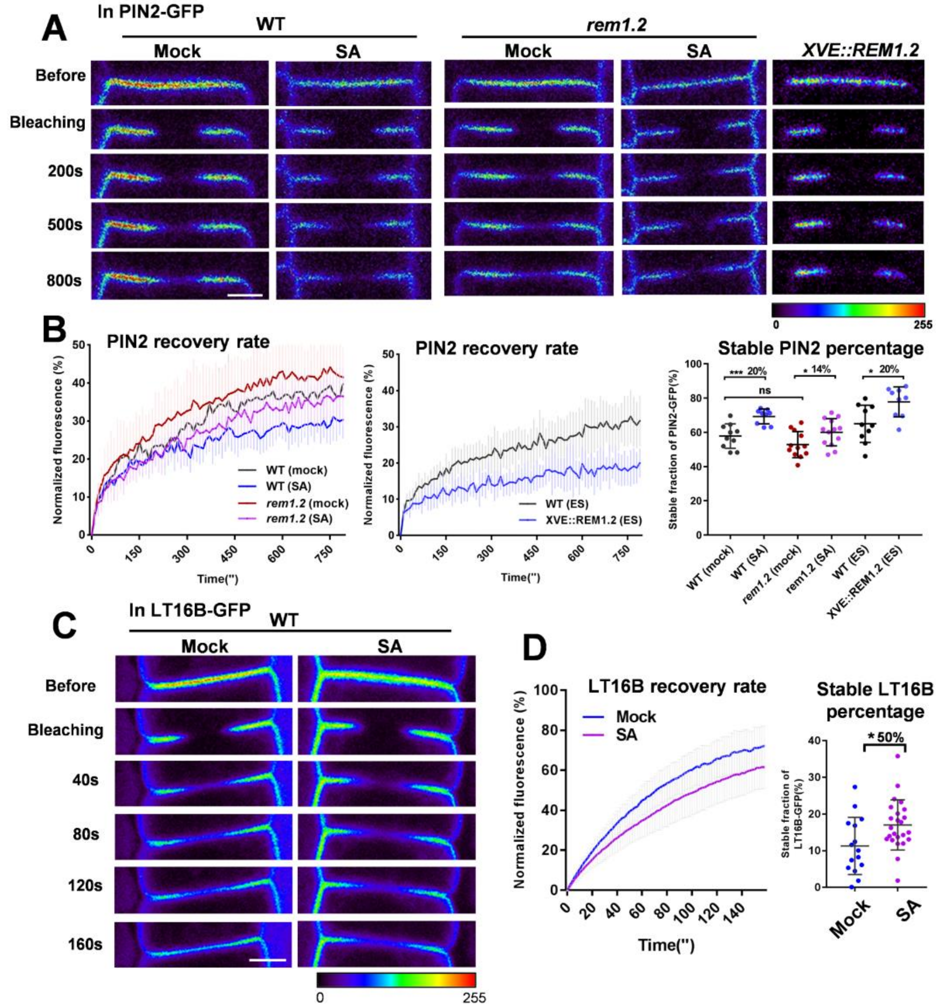

A
In PIN2-GFP
WT
Mock
SA
rem1.2
Mock
SA
XVE::REM1.2
Before
Bleaching
200s
500s
800s
0
255
B
PIN2 recovery rate
Normalized fluorescence (%)
Time('')
WT (mock)
WT (SA)
rem1.2 (mock)
rem1.2 (SA)
PIN2 recovery rate
WT (ES)
XVE::REM1.2 (ES)
Stable PIN2 percentage
Stable fraction of PIN2-GFP(%)
*** 20%
ns
* 14%
* 20%
WT (mock)
WT (SA)
rem1.2 (mock)
rem1.2 (SA)
WT (ES)
XVE::REM1.2 (ES)
C
In LT16B-GFP
WT
Mock
SA
Before
Bleaching
40s
80s
120s
160s
0
255
D
LT16B recovery rate
Mock
SA
Normalized fluorescence (%)
Time('')
Stable LT16B percentage
Stable fraction of LT16B-GFP(%)
* 50%
Mock
SA

Fig. S7 *Arabidopsis* PIN2-GFP clusters and dynamics on plasma membrane.

(A) Representative image of PIN2-GFP on the PM of root epidermal cells under VA-TIRFM (variable-angle total internal reflection fluorescence microscopy). The arrows highlight the particles representing three populations of PIN2-GFP foci at high-intensity (red arrows), medium-intensity (yellow arrows) and low-intensity (blue arrows).

(B) The moving trajectories of the highlighted high-, medium-, and low-intensity particles in (A) in 4 s time course with 0.1 s interval.

(C) Quantification of the PIN2-GFP particle diffusion coefficient for the three highlighted groups in (A). n=20 randomly selected particles from >3 images for each group.

(D-E) 4-day-old seedlings of WT and *GRF-amiR* line were continuously grown on 1/2 MS with 5μM estradiol or transferred to agar plates with 5 μM estradiol and 100 μM salicylic acid (SA) before being reoriented by 90 °. Root tip angles were measured according to the percentage of the deviated angle.

Scale bars, 5 μm (A). Error bar=S.D. in C, D, S.E.M in D and E. P-values were determined by two-tailed Student's t-test assuming equal variances (*p < 0.05, ***p< 0.001, ****p< 0.0001).

Supp. Fig. 7

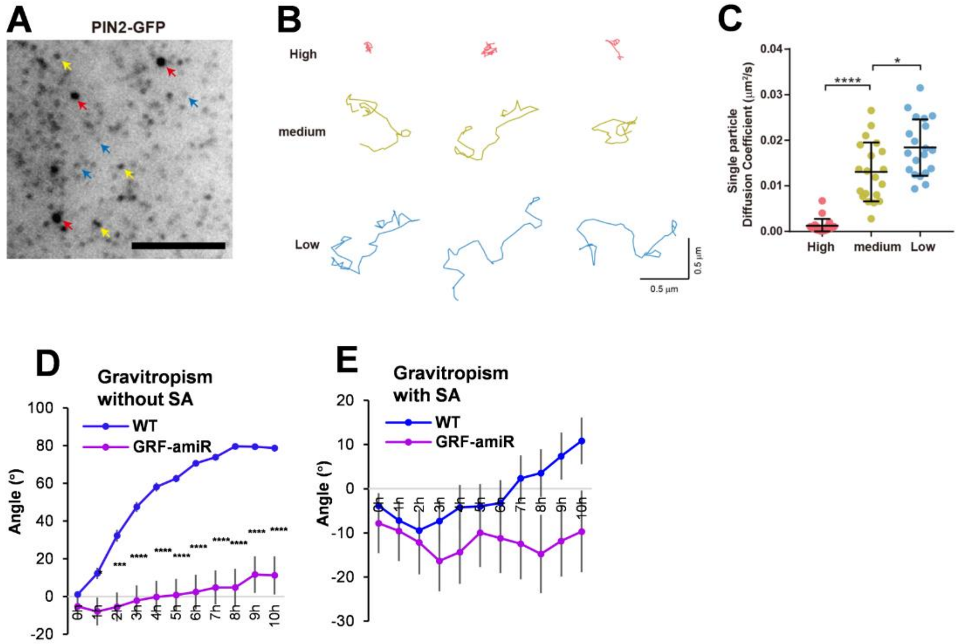

A
PIN2-GFP
B
High
medium
Low
0.5 µm
0.5 µm
C
Single particle Diffusion Coefficient (µm²/s)
0.04
0.03
0.02
0.01
0.00
High
medium
Low
****
*
D
Gravitropism without SA
WT
GRF-amiR
Angle (°)
E
Gravitropism with SA
WT
GRF-amiR
Angle (°)

Table S1 List of primers used for genotyping and qRT-PCR analysis.

	Forward primers (F) and Reverse primers (R)
REM1.1-promoter	F: 5'AGATGGTTAGAGAGGCCTGGTTTGGAAGGATGTTATTTAATAACAATTATAAAC 3'<br>R: 5'AGGAGGCCATACTAGTGGTTTCTTGTGTATTACGATTGATCAAT 3'
REM1.4-promoter	F: 5'GGGGACAAGTTTGTACAAAAAAGCAGGCTTTGTGACTCGAATTGCTATTAAAAC 3'<br>R: 5'GGGGACCACTTTGTACAAGAAAGCTGGGTTGATGTTAGGTACAACAACTAAAG 3'
CLC2 promoter	F: 5'AGCTAAGCTTGAGCTCGAATGTGTTAAGTCTTTGTTTATTTTTGTTTTAGGC 3'<br>R: 5'TTGTGATATCACTAGTCTCTCGAACACACAAAAAAAAAAAAAAAGG 3'
CLC2-gDNA	F: 5'GGGGACAAGTTTGTACAAAAAAGCAGGCTTTATGTCTGCCTTTGAAGACGATTC 3'<br>R: 5'GGGGACCACTTTGTACAAGAAAGCTGGGTTAGCAGCAGTAACTGCCTCAG 3'
DRP2B promoter	F: 5'AGCTCAAGCTAAGCTTTTTTCGCCGTCGATGCA 3'<br>R: 5'AGGAGGCCATACTAGTCGTAACTTGCACAGCGAATC 3'
DRP2B-CDS	F: 5'GGGGACCACTTTGTACAAGAAAGCTGGGTTCTAATACCTGTAAGATGATCCAGACTGTG 3'<br>R: 5'GGGGACAAGTTTGTACAAAAAAGCAGGCTTAATGGAGGCGATCGATGAGT 3'
rem1.2-T-DNA	F: 5' ATGGCGGAGGAACAGAAGATA 3' 3<br>R: 5'TACAGGTTCTGTAAAAAAATTCA 3'
NHL19-CDS	F: 5'GGGGACAAGTTTGTACAAAAAAGCAGGCTTCATGGGAGAAGGAGAAGC3'<br>R: 5'GGGGACCACTTTGTACAAGAAAGCTGGGTCCTAGACATCAACTTTACAAG3'

Table S2 Cloning Strategy.

**Name**	**Promoter**	**Vector**
pREM1.1:GFP-REM1.1	pREM1.1	pK7WGF2
pREM1.4:GFP-REM1.4	pREM1.4	pB7WGF2
35S:RFP-REM1.2	35S	pK7WGR2
pREM1.2:RFP-REM1.2	pREM1.2	pK7WGR2
pCLC2:CLC2-RFP	pCLC2	pK7RWG2
pDRP2B:DRP2B-RFP	pDRP2B	pK7RWG2
XVE::NHL19	XVE inducible	pMDC7B

Methods S1 Quantification method

i. Quantification of PIN2/REM cluster area and density: confocal pictures that were scanned by Zeiss LSM 880 (with Airyscan) with the same setting. All images were adapted to the same visualization setting and quantified by Zeiss software. PIN2 or REM1.2 signal spot without visible signal interruption was defined as an island, and the island area <0.6 μm^2 was defined as a cluster. The island area was quantified according to their areas at the apical PM of each cell.

ii. Quantification of PIN2 clustering index: PIN2-GFP signal was captured using Zeiss LSM880 with Airyscan mode under 488 nm laser excitation and 500-550 nm emission. In every individual experiment, all the samples were scanned by the same setting. PIN2 signal profile at the PM was tracked by plotting the apical membrane to generate signal values along with a series of equidistant PM automatically. The signal was automatically tracked by Image J (Plot Profile). The signal intensity of each plot was divided by the average signal intensity of each cell to generate the clustering index. At least 30 cells were quantified and more than 160 dots were automatically tracked in each cell. Thus, more than 5000 relative signal intensity was obtained in each sample. Finally, the histogram chart of the GFP signal shows the distribution of the PIN2 signal indicating the clustering index.

iii. Percentage of signal intensity value of PIN2-GFP beyond 95% confidence (outside confidence): After the quantification of PIN2 clustering index, the relative signal intensity of PIN2 within 95% confidence was defined in the mock group according to the lower and upper confidence limit (Supp. Fig. 2B). The value is smaller than the lower confidence limit or greater than the upper confidence limit (beyond 95% confidence interval) is collected, and the percentage of outside the confidence for each cell is calculated.

iv. CLC2 and DRP2B lifetime: CLC2-RFP and DRP2B-RFP single particles were captured by VA-TIRFM equipped on the Zeiss Elyra PS.1 super-resolution system. Time-lapse images up to 90s with 1s interval were acquired by using the Zeiss Alpha Plan Apochromat 100x (NA=1.46) oil objective. The deconvolution was further carried out on those acquired images to correct the background signal using Huygens Essential

(Scientific Volume Imaging). CLC2-RFP and DRP2B-RFP lifetimes were then quantified by extracting the particle kymographs in ImageJ. Lifetimes of more than 50 particles from at least three seedlings were analyzed.

v. PIN2 diffusion velocity and efficiency: The PIN2-GFP particle time-lapse images with 100ms exposure time and no interval were captured using the same microscope as CLC2 lifetime. The first frame of the time-lapse images were subjected to ImageJ plugin Trackmate for particle selection to quantify the total intensity of PIN2-GFP particles after background subtraction. Based on the preliminary analysis in ImageJ, the particle diameter was set as 0.3 μm for the following particle intensity analysis. The time-lapse images were first cropped into 5 μm × 5 μm Region Of Interests (ROIs), which were next imported to SpatTrack (Lund *et al.*, 2014) for single-particle tracking and quantifying the PIN2-GFP dynamic. The estimated particle diameter and the intensity threshold were set as 0.3 μm and 96%, respectively, for particle detection. The largest displacement between each frame was set as 0.5 μm, and the gap closing event was not allowed to ensure the precise extraction of the particle trajectories. The final trackings with more than 1.5s time course were extracted to plot the trajectories and further used for MSD quantification in SpatTrack by using the following equation:

$$\mathrm{MSD}(t) = \frac{1}{M-n}\sum_{i=1}^{M-n}\left\{\left(x(i+n)-x(i)\right)^2 + \left(y(i+n)-y(i)\right)^2\right\}$$

in which the x, y represents the particle location, M indicates trajectory length with respect to the image frame, and n is the frame number corresponding to t. The Diffusion Coefficient was further analysed by using the equation MSD(t)=4Dt in which D represents Diffusion Coefficient and MSD(t) was obtained by fitting the original MSD curve with the browning diffusion model. For each treatment, more than 30 ROIs from at least ten time-lapse images taken from more than three seedlings were analysed.

vi. Quantification of asymmetric PIN2 distribution: The images were imaged by Leica confocal and exported to TIFF format, and PIN2 signal profile in the upper and lower sides was measured by plotting a line along the epidermis cells (with the same number of cells of upper and lower side) by Image J. Then the area of signal profile images was calculated in GraphPad by "area under curve", and the ratio of lower side compared with upper side was counted.

vii. Quantification of FRAP data: FRAP was performed by Leica SP8 confocal. Before bleaching, GFP signaling was acquired by using 488 nm laser excitation and 500-550 nm emission. Similar ROIs of apical PIN2 or LT16B were selected and photobleached by scanning with the 488 nm argon laser at 100% laser power. After bleaching, the image was captured by same setting with pre-bleaching (80 frames at 10 s intervals). The fluorescence recovery of the region of interests (ROIs) was measured by Image J. Quantification of FRAP data was referred to the previous study (Mahen *et al.*, 2011; George *et al.*, 2015).

viii. Co-localization value: Images from randomly seleted ROIs were acquired by Zeiss LSM880 with Airyscan mode. Co-localization was determined by ImageJ software with Pearson correlation coefficient

References

George J, Soares C, Montersino A, Beique JC, Thomas GM. 2015. Palmitoylation of LIM Kinase-1 ensures spine-specific actin polymerization and morphological plasticity. *Elife* **4**: e06327.

Lund FW, Jensen ML, Christensen T, Nielsen GK, Heegaard CW, Wustner D. 2014. SpatTrack: an imaging toolbox for analysis of vesicle motility and distribution in living cells. *Traffic* **15**(12): 1406-1429.

Mahen R, Jeyasekharan AD, Barry NP, Venkitaraman AR. 2011. Continuous polo-like kinase 1 activity regulates diffusion to maintain centrosome self-organization during mitosis. *Proc Natl Acad Sci U S A* **108**(22): 9310-9315.
